# TOP2 and NOS2 Orchestrate the Generation of DNA Breaks to Promote Colitis Cancer Initiation

**DOI:** 10.3390/cancers18101519

**Published:** 2026-05-08

**Authors:** Ting-Kang Chang, Shiu-Ling Li, Anne-Cécile Brunac, Jia-Jun Huang, Yen-Hsiu Yeh, Pierre Brousset, Jean-Marc Egly, Tsai-Kun Li

**Affiliations:** 1 Department and Graduate Institute of Microbiology, College of Medicine, National Taiwan University, No. 1 Jen-Ai Road Section 1, Taipei 10051, Taiwan; d02445001@ntu.edu.tw (T.-K.C.); lightenight@gmail.com (S.-L.L.); junjune217@gmail.com (J.-J.H.); bromoyeh2004@gmail.com (Y.-H.Y.); 2 Department of Pathology and Cytology, Institut Universitaire du Cancer de Toulouse-Oncopole, 1 Avenue Irène Joliot-Curie, 31059 Toulouse, France; brunac.annececile@iuct-oncopole.fr; 3 Laboratoire d’Anatomie Cytologie Pathologiques, IUCT-Oncopole, 1 Avenue Irène Joliot-Curie, 31059 Toulouse, France; brousset.p@chu-toulouse.fr; 4 Department of Functional Genomics and Cancer, Institut de Génétique et de Biologie Moléculaire et Cellulaire (IGBMC), CNRS/INSERM/University of Strasbourg, BP 163, Illkirch Cédex, 67404 Strasbourg, France; egly@igbmc.fr; 5 Centers for Genomics and Precision Medicine, National Taiwan University, Taipei 10055, Taiwan; 6 Advanced Biomedical Research Center, Taiwan Centers of Excellence, National Taiwan University, Taipei 10672, Taiwan; 7 Development Center for Biotechnology, Taipei 11571, Taiwan; 8 Department and Graduate Institute of Biochemistry, National Defense Medical Center, Taipei 11490, Taiwan

**Keywords:** inflammation, DNA damage response, colitis-associated colorectal cancer, DNA topoisomerase, nitric oxide synthase, inflammatory bowel disease, knockout mice

## Abstract

**Background:** Inflammatory bowel disease (IBD), including ulcerative colitis (UC) and Crohn’s disease (CD), increase colorectal cancer (CRC) risk. **Methods:** Mouse IBD and CRC models with a combination of pharmacological, knockout and knock-in approaches was employed to analyze the involvement of TOP2s and NOS2 in CRC tumorigenesis. Key pathologies, such as inflammatory and neoplastic scores, were examined by immunohistochemical assays. **Results:** In colon tissues from acute, chronic colitis and CRC mouse models and from CD patients, the biomarkers γH2AX and 53BP1_pS25/S29_ of DNA breaks (mainly representing DSBs) accumulated, alongside increases in topoisomerase II (TOP2) and nitric oxide synthase 2 (NOS2). Genetic ablation of NOS2 (*Nos2^-/-^*) or TOP2β (*Top2β^f/f^*) as well as pharmacological inhibition with ICRF-193 (a TOP2 inhibitor) or PTIO (a NO scavenger) reduced DSB formation and disease severity. Consistently, *Nos2^-/-^*, or ICRF-treated, mice exhibited decreased tumor burden. DSBs and tumor accumulation were pronounced in the distal colon, mirroring human CRC distribution. While ICRF-193 suppressed tumor growth, *Top2β^f/f^* deficiency (with a compensatory TOP2α upregulation) enhanced tumor development, indicating potential roles for TOP2 isozymes in tumor formation and progression. **Conclusion:** Collectively, these findings identify the cooperative action of TOP2 and NOS2 in driving DSBs, highlighting a potential therapeutic target in inflammation-associated CRC.

## Simple Summary

Our experimental data evidently offer underlying mechanism(s) and clinical relevance for the elevated risk of colitis-associated colorectal cancer (CRC) linked to inflammatory bowel disease (IBD). We first found γH2AX accumulation in tissue samples from Crohn’s disease (CD) patients and mouse models for IBD and CRC, and detected 53BP1_pS25/S29_ (another DSB marker) in inflamed cell lines. Both pharmacological inhibitions of TOP2 and NO production reduced γH2AX and 53BP1_pS25/S29_ levels caused by inflammation. The importance of the TOP2β isozyme and NOS2 in the generation of colitis-associated CRC is also supported using genetically deficient mice. From the realization of the respective roles of TOP2β and TOP2α in cancer initiation and progression and our understanding of pathogenic mechanism(s), our studies thus offer precision targets and corresponding intervention strategies.

## 1. Introduction

Ulcerative colitis (UC) and Crohn’s disease (CD) belong to a set of chronic and idiopathic immunological disorders of the gastrointestinal tract, mainly limited to the colon, and are referred to as inflammatory bowel diseases (IBDs). A relationship between inflammation and cancer is exemplified by the increased risk for colorectal cancer (CRC) in patients with IBD, particularly in those with UC. The incidence rates of cancer in patients with UC are approximately 8% and 18% at 20 and 30 years old, respectively [1,2]. With 1.2% of CRC occurring with CD, this represents a 20 times greater risk of occurrence [3]. The clinical importance of IBD is further evidenced by the observation that CRC accounts for >10% of all deaths in patients with IBD. IBD-related deaths account for only 1–2% of all CRC cases (i.e., an estimated 5.5–11 times greater mortality rate than patients with CRC without IBD) [4]. Moreover, the age at diagnosis of CRC associated with IBD is >15 years younger than that of sporadic ones [2]. In addition to tumor-promoting inflammation, “gene instability and mutation” have also been attributed as one of the two enabling characteristics of cancer that assist cells in the transition from normal to neoplastic [5,6]. Of note is the fact that a population-based study suggested a potential hormonal influence in IBD pathogenesis, reporting a higher UC incidence in men compared to women after the age of 45 [7].

Cancer is a complex genetic disease with unique hallmarks that could offer therapeutic interventions. In the last two decades, an advanced understanding of cancer genetics, epigenetics and molecular biology has revealed the functional significance of genomic instability in tumorigenesis, particularly in the hyperplasia–adenoma–carcinoma sequence of CRC [8]. Indeed, CRC is often associated with DNA damage and repair, classified as: (i) chromosome instability (CIN, 80–85%) with a prevalence on somatic copy number changes and (ii) microsatellite instability (MIN/MSI, 15–20%) with a higher mutation rate [6,9]. Although the origin of MIN could be largely attributed to DNA, base lesions and mismatch repair (MMR) deficiency in CRC, the origin of CIN remains largely unknown. Nevertheless, it seems that DNA double-stranded breaks (DSBs) and the corresponding DNA damage response (DDR) could be one major initiation source of CRC. This notion is supported by observations that: (i) elevated p53 expression in dysplastic crypts might serve as a good biomarker in the early stages of UC-associated colon tumorigenesis [10]; (ii) radical-induced p53 activation was observed during chronic inflammation parallel with cancer development [11]; and (iii) higher γH2AX accumulation, a DSB marker [12], was detected in UC [13] and could be used to predict a poor diagnosis in CRC [14]. Notably, γH2AX and phosphorylated p53-binding protein (53BP1_pS25/S29_) are considered as DSB markers [15]. However, the potential mechanistic link between inflammation, DDR, and genomic instability requires further studies, such as single-cell RNA sequencing analysis.

Therefore, it appears that acquisition of cancerous propensities is due to certain mutations. Moreover, pro-inflammatory cues also facilitate the transformation from normal to cancerous tissue. Both acute and chronic colitis are key risk factors for CRC [16,17]. In addition to the tumorigenesis of primary cancer, there is a relationship between DNA topoisomerase II beta (TOP2β) with the generation of secondary malignancies during etoposide (Etop) therapy [18,19]. Two TOP2 isoforms, TOP2α and TOP2β, have been found in human cells. Because of their ability to relax DNA (with a DNA cleavage and re-ligation mechanism), they are essential for DNA processes such as transcription, replication and cellular proliferation. Drugs, such as Etop, target TOP2 to induce DNA breaks, γH2AX accumulation, mutations and cell death [18]. TOP2α is highly expressed in cancerous cells and is considered as the primary target for drug efficacy. Drug-induced TOP2β-mediated DNA breaks are associated with adverse effects, such as therapy-related secondary malignancies and cardiotoxicity [19]. Among these two isozymes, TOP2β is more sensitive to radicals, and together they contribute to DNA damage and mutations [17,19,20,21].

During tumorigenesis, pro-inflammatory radicals (reactive nitrogen/oxygen species, RNOS) are recognized as mutagenic sources and potential carcinogenic factors [22,23]. In chronic colitis, the production of these free radicals, such as hydrogen peroxides (H_2_O_2_) and nitric oxide (NO), may directly contribute to DNA damage, thus subsequently linking inflammation and RNOS-mediated DNA damage responses to dysplasia and cancer progression [24]. Notably, NO is generated through a stimulated expression of inducible NO synthase (iNOS/NOS2) during inflammation [22]. NO has been reported to nitrosylate macromolecules, such as nitrosylation of cysteine on proteins, while our previous results have shown that suitable TOP2 thiol modifications induce the formation of TOP2-cleavable complex (TOP2cc), DNA breaks and subsequent γH2AX detection. In patients with UC, the colon is exposed to nitrosylated and oxidative stresses [25,26], which could in turn activate DDR, particularly DSB repair pathways [27]. It would be interesting to investigate the direct connection between inflammation, NO, and TOP2 during colitis conditions, as well as potential contributions of these molecules to the development of colitis-associated tumorigenesis.

In this study, we modeled acute and chronic colitis as well as tumor development in mouse colons using dextran sulfate sodium (DSS) and/or azoxymethane (AOM). We identified a key role of TOP2β in DSB formation that is marked by γH2AX detection in both mouse colons and CD patient samples. Colon-specific deletion of *Top2β*, *Nos2* knockout or treatment with TOP2-antagonizing inhibitors (ICRFs) in mice all led to reduced DSB and tumor formation. We also observed that the locations of the tumors coincided with γH2AX accumulation, as observed in the distal segment of the mouse colons. Notably, tumor localizations corelated with regions of high γH2AX accumulation, particularly in the distal colon. Mechanistically, we showed that NO in combination with TOP2 promotes γH2AX and 53BP1_pS25/S29_ accumulation in HCT116 cells and induces DSB in vitro in the presence of recombinant TOP2 isozymes.

## 2. Materials and Methods

### 2.1. Ethics Statement

All mouse experiments were approved by the Institutional Animal Care and Use Committee (IACUC) of the National Taiwan University College of Medicine. The human study was approved by a local Ethics Committee (Comité de Protection des Personnes Sud-Ouest et Outremer II) of France.

### 2.2. Mouse Strains and Cell Lines

Wild-type (WT) C57BL/6Jnarl and B6.Cg-Tg(Vil-cre)20SyNci strains [28] were obtained from the National Applied Research Laboratory (Academia Sinica, Taipei, Taiwan) and Frederick National Laboratory (Product #01XE7, Frederick, MD, USA) respectively. The B6.129P2-*Nos2 ^tm1Lau^*/J and 129S-*Top2β^tm2Jcw^*/J mutant strains were obtained from the Jackson Laboratory (Product #002609 and #008396, Bar Harbor, ME, USA). For consistency, all mutant mice with a 129S background were backcrossed with WT mice for at least four generations. All imported mice were acclimatized for more than one week before the experiments, and mouse experiments were conducted in a pathogen-free facility. The RAW264.7 cell line was kindly provided by Prof. S-C Miaw (National Taiwan University, Taipei, Taiwan). The HCT116 colorectal cancer cell line was originally obtained from ATCC.

### 2.3. Colitis and Cancer Disease Models and Treatments

#### 2.3.1. Acute and Chronic Colonic Colitis (DSS-UC) Mouse Disease Models

Acute and chronic colitis models were established as reported [29]. Briefly, 1.0–5.0% (*w*/*v*) DSS (MW 36,000–50,000; MP Biomedicals, Santa Ana, CA, USA) samples were prepared in distilled water and sterilized by a 0.22 μm filter. The DSS samples were placed in water bottles, allowing the mice to drink in an ad libitum fashion (4–5 mice per group, >3 independent experiments). For the acute model, 7-week-old male mice were given 2.5% DSS for 7 days. In addition, 6- to 8-week-old female mice were alternatively provided with 1.0, 3.0 and 5.0% DSS for 5 days. For the chronic model, 8-week-old male mice were given 3 cycles of 2.5% DSS for 7 days on weeks 2, 5, and 8, followed by 2 weeks of water recovery (70 days). The negative control groups were provided with only distilled water. The mice’s body weight, water consumption, and disease activity index (DAI) were monitored during the treatment. After treatment finished, the mice were sacrificed through cervical dislocation, and colons were harvested, longitudinally opened, and fixed in 10% (*v*/*v*) neutral buffered formalin for 24–48 h. The colonic images were captured using a camera (Product # DSC-QX100, Sony, Tokyo, Japan) and were then processed in a Swiss-roll manner [30], embedded in paraffin, and mounted for histo-molecular analyses.

#### 2.3.2. The Two-Stage AOM/DSS Colon Cancer Disease Model

The colon cancer disease model was established as previously described [31]. Briefly, 8-week-old male mice (4–5 mice per group, >3 independent experiments) were administered 10 mg/kg azoxymethane (AOM, Sigma-Aldrich, Saint Louis, MO, USA) via intraperitoneal (IP) injection in the first day of week one. Then, the mice were treated with three cycles of 2.5% DSS for 7 days on weeks 2, 5, and 8, followed by two weeks of water recovery (70 days). For the double-gene-knockout mouse model, the cycles were adjusted to 2.0% DSS for 5 days with an additional one week of water recovery after the last cycle (77 days). The negative control groups were provided with solely distilled water. After being sacrificed, the colons were longitudinally opened, fixed, and stained with Alcine blue. Alcine blue stained the mucin of epithelial origin, which facilitated the visualization of tumor(s). The tumor numbers of the distal, middle, and proximal segments were counted through direct visual assessment. The tumor diameters were measured, and corresponding volumes (V_T_) were then calculated using the formula V_T_ = 4/3* πr^3^ (r = the radius of the tumor). After that, the colon tissues were also processed in a Swiss-roll manner and embedded in paraffin for further analyses.

#### 2.3.3. Drug Treatments

Etoposide (Etop), dexrazoxane (ICRF-187, Abcam, Cambridge, UK) and ICRF-193 (Enzo Incorporation, Serangoon, Singapore) were used, wherein appropriate drug dosages were administered accordingly [19]. Briefly, drugs and control DMSO (Ctrl) were administered via IP injection. As a positive control for DNA break, Etop (40 mg/kg) was injected twice on day 0 and 4. ICRF-187 (15 mg/kg) or ICRF-193 (2.5 mg/kg) were administered one day before and on the third day during DSS treatment.

### 2.4. Generation of Gene-Defective Mouse Strains and Genotyping

#### 2.4.1. Cross-Breeding

The *Top2β*-knockout mouse strain (*vil-Cre Top2β^f/f^*; B6; 129S *Top2β^tm2.1(vil-cre)Jcw^*/J) was generated through two generations of breeding crosses between B6.Cg-Tg(*Vil-cre*)20SyNci (LASP Mouse Repository; a kind gift from Prof. S. Takahashi, Japan; *vil-Cre*) [28] and 129S-*Top2β^tm2Jcw^*/J (Jackson Lab; kindly provided by Prof. L.-F. Liu, USA; *Top2b^f/f^*). The *Nos2* and the *Top2β* double-knockout mice were generated through four generations of breeding crosses between the *Nos2^-/-^* (B6. 129P2-*Nos2^tm1/Lau^*/J) and *vil-Cre Top2β^f/f^* strains. In the first two generations, the *Nos2^-/-^* mice were first crossed with the *vil-Cre* or *Top2b^f/f^* strain, leading to the generation of two mutant strains without the *Nos2* gene (i.e., *Nos2^-/-^ vil-Cre* and *Nos2^-/-^ Top2β^f/f^* strains). Next, these two mutant strains were crossed with the *vil-Cre Top2β^f/f^* strain to generate a double-knockout mouse (*Nos2^-/-^ vil-Cre Top2β^f/f^*; B6; 129S-*Top2β^tm2.1(vil-cre)Jcw^ Nos2^tm1Lau^*/J).

#### 2.4.2. Mouse Genotyping

The genotypes of the mice were determined by PCR analyses [32,33]. Briefly, 3–6 mg of tissue was collected from the tails, and genomic DNA were extracted using a MyTaq Extract Kit (Bioline, Hong Kong, China). A total of 1 μg of extracted DNA was then added to the MyTaq HS Red Mix with 10 mM of the appropriate primers for a final volume of 20 μL. The PCR reactions were conducted using a SensoQuest LabCycler 96 thermocycler, and the DNA samples were separated through a 2–3% agarose gel in 0.5% Tris–phosphate–EDTA (TPE) buffer. After staining the gels, the corresponding DNA fragments were visualized and imaged using a Dolphin-Doc Plus imaging system. All the primers are listed in Supplementary Table S1.

### 2.5. Hematoxylin and Eosin Staining and Immunohistochemistry Analyses

#### 2.5.1. H&E Staining

Formalin-fixed and paraffin-embedded colon tissues were dissected into a thickness of 4 μm and mounted on slides. After drying for 1 day, the tissue was stained with H&E (Leica, Wetzlar, Germany) accordingly [34]. Tissues were examined under an Eclipse 80i microscope with a Ds-Fi1 digital camera (Nikon, Tokyo, Japan).

#### 2.5.2. IHC Mouse Tissue Staining

IHC was performed on the mouse tissues as previously described [35]. Tissue samples were prepared, mounted on immune-coated slides (Muto Pure Chemicals, Tokyo, Japan), and deparaffinized, and rehydrated antigens were retrieved through two approaches: (i) antigens of caspase 3, Ki-67, γH2AX, TOP2α and TOP2β were exposed by boiling the tissues in 10 mM sodium citrate buffer (pH 6.0) at 95 °C for 15 min; (ii) antigens of NOS2 and F4/80 were restored through digestion of 200 mg/mL of trypsin at 37 °C for 15 min. After retrieval, the tissues were incubated in 3% H_2_O_2_ at room temperate (RT, 15 min) to quench the endogenous peroxidase activity and then blocked in 5% bovine serum albumin (BSA, RT, 60 min). The epitopes of interest were detected using rabbit γH2AX monoclonal antibodies (mAbs, 1:400 dilution, Genetex, Irvine, CA, USA), CASPASE3^N175^ mAbs (1:200, CST, Danvers, MA, USA), Ki-67 mAbs (1:400, Abcam), NOS2 polyclonal antibodies (pAbs, 1:1200, Millipore, Saint Louis, MO, USA), TOP2α pAbs (1:300, Abcam), TOP2β pAbs (1:500, Santa Cruz Biotechnology, Dallas, TX, USA) and mouse F4/80 mAbs (1:200, Cedarlane, Burlington, NC, USA). These antibodies were diluted in either phosphate-buffered saline (PBS) for cellular membrane markers (e.g., F4/80) or 0.2% (*v*/*v*) PBS/Tween-20 (PBS-T) for intracellular markers (e.g., γH2AX, CASPASE3^N175^, Ki-67, NOS2, TOP2α, TOP2β). Primary antibody incubation was conducted at 4 °C for 16 h. The next day, the tissues were washed thrice with PBS or PBS-T at RT for 10 min. The tissues were then probed with the appropriate secondary antibody (either a rabbit universal pAbs from Nichirei Biosciences (Tokyo, Japan) or a mouse IgG-Fc pAbs from Bethyl Laboratories, Montgomery, TX, USA) for 60–120 min at RT. Subsequently, a chemical chromogenic reaction was performed using the DAB substrate mix (ImmPACT Bio, West Hills, CA, USA). The respective durations of DAB exposure for different biomarkers were as follows: γH2AX (120 s), CASPASE3^N175^ (10 min), Ki-67 (120 s), NOS2 (30 s), F4/80 (120 s), TOP2α (60 s) and TOP2β (20 s). The tissues were counterstained with hematoxylin for 30–40 s. The slides were dehydrated and mounted on a cover glass. Representative images were obtained using an Eclipse 80I microscope (Nikon, Japan) equipped with a DS-Fi1 CCD camera.

#### 2.5.3. IHC Human Tissue Staining

The IHC of human tissue samples was provided and stained by the Pierre Brousset Laboratory. Staining was performed using the automated slide stainer Discovery ULTRA (Roche Diagnostics, Hong Kong, China). Briefly, the slides were heat-treated for antigen retrieval using CC1 buffer (pH 8.0) and incubated with primary rabbit polyclonal anti γH2AX antibody (Phospho-S139, Abcam). The epitopes were subsequently visualized using an OmniMap DAB Anti-Rb Detection Kit (Roche Diagnostics), and the nuclei were counterstained with hematoxylin. For interpretation, the slides were evaluated using light microscopy.

#### 2.5.4. IHC Quantitation

IHC signals, as visualized by the intensity of brown staining compared to the controls, were quantified using the ImageJ software (version 1.46) and/or manual counting. The ImageJ approach was conducted using the color deconvolution plugin to separate the DAB signals from the remainder. The signals were converted into black pixels with thresholds of 150 for F4/80, 200 for γH2AX, 150 for Ki-67, 150 for NOS2, 180 for TOP2α, and 150 for TOP2β. The signal intensities were then measured as the area fraction of “black pixels” relative to “the total number of pixels”. For signal loss during epithelial erosion, and to complement the ImageJ approach, manual counting was used to ensure signal detection. The manual approach was used to count at least 100 cells per image (more than 2 images per sample). In both the approaches, at least two images were determined for each tissue sample.

### 2.6. Pathological, Disease and Neoplastic Scoring

#### 2.6.1. Disease Activity Index

Mouse DAIs were scored with stool consistency (score 0–3) and fecal blood (score 0–3) [29]. Stool consistency was scored as follows: 0, normal; 1, soft but still formed; 2, very soft; and 3, diarrhea. Fecal blood had a score of 0 for negative hemoccult, 1 for positive hemoccult, 2 for visible blood traces in the stool, and 3 for rectal bleeding. A Hemoccult SENSA Developer kit (Beckman Coulter, Brea, CA, USA) was used to detect fecal blood beyond the visible range.

#### 2.6.2. Inflammatory Score

Inflammatory scores were determined by H&E images of epithelial erosion (score 0–2), crypt abscess (score 0–2), mucin depletion (score 0–2), and immune cell infiltration (score 0–3) [36]. Epithelial erosion was defined as a score of 0 for normal tissue, 1 for focally stripped erosion, and 2 for ulcers or granulation tissue. The scores of crypt abscess were defined as 0 for no abscess, 1 for probable crypt destruction, and 2 for ulcers or no observable crypt. Mucin depletion was scored 0 as none, 1 for moderate, and 2 for severe depletion. Immune cell infiltration was scored 0 as none, 1 as scattered, 2 as moderate, and 3 as dense infiltration of immune cells in the mucosa and submucosa regions.

#### 2.6.3. Neoplastic Score

The neoplastic score was calculated as described previously [37]. Briefly, the score was given as no neoplasia (score 0), mild neoplasia with aberrant crypt foci (ACF, score 1), extensive ACF with small gastrointestinal neoplasia (GIN, score 2), low-grade adenoma (score 3), high-grade adenoma (score 3.5), adenocarcinoma invading into or through the muscularis mucosa (score 4), and carcinoma invading into or through the muscularis propria and serosa (score 5). High-grade adenoma normally has a high nuclear/cytoplasm (N/C) ratio, less mucin secretion, less inter-glandular stroma formation, and more cribriform (sieve-like) space compared with low-grade adenoma. The N/C ratio was determined based on the purple/pink color in the H&E images.

### 2.7. Immunofluorescence Analysis and Quantification

#### 2.7.1. Immunofluorescence Analyses

Immunofluorescence (IF) analysis of HCT116 cells was performed as previously described [38]. Briefly, 1 × 10^5^ HCT116 cells were seeded into each well of a 6-well plate on a heat-disinfected cover glass. After overnight cultivation in a CO_2_ incubator. The cell culture medium was removed. The cells were washed with 1X PBS buffer. To promote DNA damage, 50 μM Etop (1 h), 5.0% DSS (4 h), or 100 μM GSNO (4 h) were added to the cells for an appropriate amount of time. To test for the presence of NO or TOP2 in the DNA damage process, 50 μM c-PTIO, 20 μM ICRF-193, or 100 μM ICRF-187 were added to the cells as a co-treatment. The cells were separated from the media after 1–4 h of treatments. The immunofluorescence analysis was conducted by first fixing the cells with 4% paraformaldehyde in PBS at RT (20 min). Next, the cells were permeabilized with 0.2% Triton-X100 (50 rpm, 5 min). The non-specific sites were blocked with 10% BSA (RT, 60 min). The epitopes of interest were detected using rabbit γH2AX mAbs (1:400 dilution, Genetex) or rabbit 53BP1_pS25/S29_ pAbs (1:200 dilution, Bioss, Woburn, MA, USA). Primary antibody incubation was conducted at 4 °C for 16 h. The next day, the cells were washed thrice with PBS-T at RT and 50 rpm for 5 min. The cells were then probed with goat anti-rabbit Alexa Fluor 488 antibody (1:500 dilution, Invitrogen, Hong Kong, China) at RT and 50 rpm for 60 min. The antibody was removed by washing the cells thrice with PBS-T at RT and 50 rpm for 5 min. The cover glass was subsequently placed on a silane-coated slide (Muto Pure Chemicals, Tokyo, Japan) and mounted using a fluoroshield with DAPI (Genetex). Representative images were captured using an Eclipse 80I microscope (Nikon, Japan) equipped with a DS-Fi1 CCD camera.

#### 2.7.2. IF Signal Quantification

Cellular fluorescence signals were quantified through ImageJ as the total immunofluorescence (TIF) of each cell [38]. For example, the cell of interest was first measured using its fluorescent area and mean intensity. These two parameters were then multiplied to obtain the integrated density. The integrated density was corrected by removing the background readings. Overall, corrected total cell fluorescence was calculated by the following formula: “TIF = integrate density − (area of selected cells × average of fluorescent intensity from background readings)”. A minimum of ten cells were counted in each group. Values were analyzed using the GraphPad Prism software (Version 6).

### 2.8. Expression and Purification of Recombinant Proteins

Recombinant human TOP2α/β proteins were prepared using a yeast system. The TOP2α (YEpWob6) and TOP2β (YEphTOP2β) plasmids resulting in the expression of the nearly full-length TOP2α (the first 28 of the 1531 amino acids were replaced by the first five codons of yeast TOP2) [39,40] and full-length TOP2β [41] were kindly provided by Drs. James C Wang and Leroy F. Liu. The expression of recombinant human TOP2s (hTOP2s) in yeast from a galactose-inducible GAL1 promoter and the purification were carried out as previously described [39]. These plasmids were transformed into protease-deficient yeast BCY123. A single colony of the yeast strain was isolated from a CSM-U plate. The strains were then inoculated and amplified in the CSM-U broth with shaking in a 30 °C incubator. Subsequent stimulation of the expression and purification of the two isozymes were performed using cation-exchange chromatography. To lyse the cells, 1 mg/mL of glass beads was added to the yeast suspension, followed by vortexing in a cycle of 30 s burst and 30 s rest on ice 13 times. After centrifugation, the supernatant was loaded into the chromatography column packed with the Bio-RexTM 70 cation-exchange resins (Bio-Rad, Hong Kong, China) to allow the liquid to drain through (i.e., flow-through). The column was cleaned by washing three times with resuspension buffer, and then the proteins were eluted with elution–resuspension buffer mixes containing different NaCl concentrations (i.e., 100, 250, 330, 360, 400, 500, and 1000 mM). Different fractions of the protein eluents were analyzed using Coomassie Brilliant blue gels. The fractions containing the hTOP2α/β protein bands were collected for dialysis. The hTOP2α/β samples were further concentrated using a Vivaspin 20 mL, 100 KDa molecular weight cut-off column device (Sartorius, Göttingen, Germany) and then stored in appropriate storage buffers at −80 °C.

### 2.9. DNA Cleavage Assays and Quantitation of DNA Breaks

#### 2.9.1. Circular and Linear DNA Cleavage Assays

The DNA cleavage assay was performed using either circular or HindIII-linearized pRYG plasmid [42]. The 1X reaction buffer was prepared with 40 mM Tris-HCI (pH 7.5), 100 mM KCI, 10 mM MgCl_2_, 0.5 mM DTT, 0.5 mM EDTA, 30 mg/mL BSA, and 0.2 mM ATP. Briefly, the reaction mixture was prepared with 100–200 ng pRYG, TOP2 proteins (with the indicated molar ratios of DNA), 50 μM Etop, and GSNO (2-fold dilutions from 500 to 3.9 μM) in 1X reaction buffer. DNA cleavage reaction was conducted by incubating the reaction mixture at 37 °C for 30 min. Next, the final concentrations of 1 mg/mL proteinase K and 1% SDS were added. The reaction was terminated by further incubating the mixtures at 37 °C for 60 min. After the assays, TOP2 protein was removed through one round of phenol/chloroform extraction. This step ensured that all the TOP2-bound DNA was released into the liquid samples. The samples were loaded onto a 1% agarose gel in 0.5% Tris–phosphate–EDTA (TPE) for analyses. The DNA fragments were stained with ethidium bromide, visualized under UV light, and photographed using a CCD camera.

#### 2.9.2. Circular DNA Cleavage Assay Quantification

The extent of nicked (SSB) and linearized (DSB) DNA bands was quantified through Image J. The background value of the DNA alone (0%) was removed through subtraction. The remaining values were then normalized against the values of the corresponding control groups of TOP2α or TOP2β alone (as 100%).

### 2.10. Statistical Analyses and Data Availability

#### 2.10.1. Statistical Analyses

All statistical analyses and graph presentations were conducted using the GraphPad Prism 6 software. For the animal experiments, the data were collected from all mice. For the DNA and molecular experiments, the data were collected from at least three repeats. All the inflammation and neoplastic scores have been verified by expert pathologists with written reports. Statistical analyses were conducted using either a *t*-test for one numerical factor in two groups, a one-way analysis of variance (ANOVA) for one numerical factor in several groups, or a two-way ANOVA for two numerical factors. The quantitative results were assumed to follow a Gaussian (normal) distribution, and analyses were determined based on data properties. Either a paired or unpaired approach was chosen for the groups with either equal or unequal variance. The Tukey method was used to compare the means among the groups. The Sidak method was used to compare the means of independent groups. *p* values of ≤0.05 (*), ≤0.01 (**) and ≤0.001 (***) are accounted as being of statistical significance.

#### 2.10.2. Data Availability

This research work and data are available from the corresponding author upon reasonable request. All the data needed to evaluate the conclusions of the paper are presented in the paper or the online Supplementary Materials.

## 3. Results

### 3.1. Detection of γH2AX in Epithelial Cells from the DSS-Induced UC

The presence of γH2AX, a key marker of DSB, was examined in the colon tissue of mice treated with increasing doses of DSS, a known inducer of inflammation [43,44]. Within 5 days, DSS-treated female mice developed acute colitis, as indicated by dose-dependent weight loss and colon length shortage (Supplementary Figure S1A,B). The severity of inflammation was assessed based on epithelial erosion (Ee), crypt abscess (Ca), mucin depletion (Md), and immune cell infiltration (Ii) [45], revealing a clear DSS dose-dependent increase in the inflammatory score (Supplementary Figure S1C).

**Figure 1:**
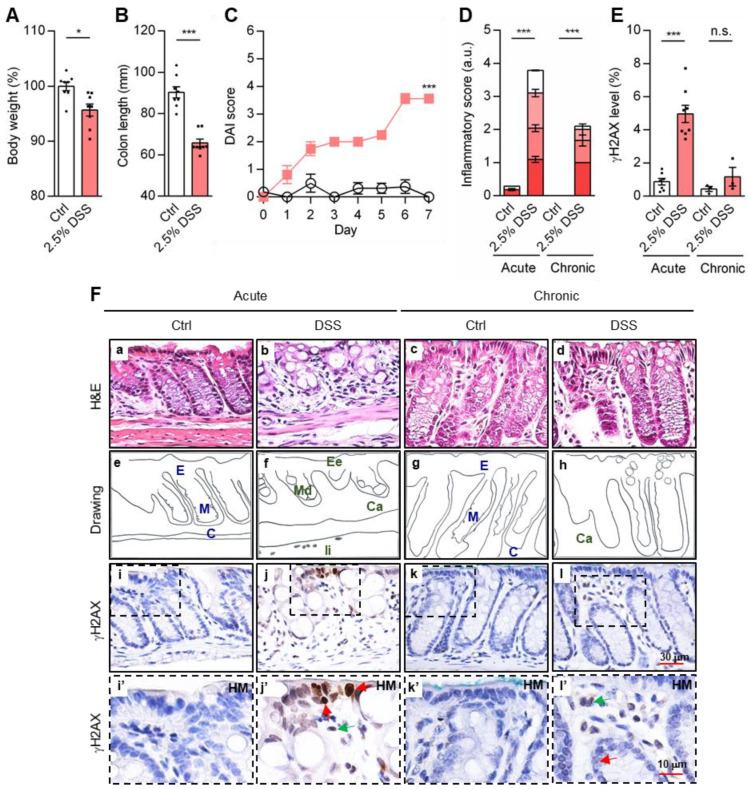
Detection of γH2AX and pathological features in the DSS-UC mice. Male mice, 7 weeks old, were treated with 2.5% DSS for 7 days to induce acute colitis (*n* = 8). (**A**) Body weight (2.5% DSS, 95.7 ± 1.1%), (**B**) colon length (Ctrl, 90.3 ± 2.8 vs. 2.5% DSS, 65.8 ± 1.9 mm) on the 7th day, and (**C**) daily DAI scores were measured according to described criteria. Circle (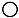): Ctrl, H_2_O alone; Square (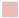): 2.5% DSS. In the chronic colitis model, male mice were administered 3 cycles of 2.5% DSS, followed by 2 weeks of recovery, to induce inflammation. (**D**) The inflammation score (colitis, acute 3.8 ± 0.2 vs. chronic 2.1 ± 0.4 a.u.) and (**E**) γH2AX levels, as a percentage of area fraction, in the acute (5.0 ± 0.5%; two repetitions, *n* = 7–8) and chronic groups (1.2 ± 0.6%; one repetition, *n* = 3). (**F**) H&E (upper panels **a**–**d**; corresponding drawings in panels **e**–**h**) staining of colon tissues revealed a normal epithelial layer (E), mucin (M) and crypt structures (C) in the control tissue samples (Ctrl). In parallel, IHC using γH2AX antibodies (panels **i**–**l**, and with high-magnification HM panels **i’**–**l’**) determined the γH2AX level. In acute and chronic DSS-treated colon specimens, the corresponding pathologic structures of epithelial erosion (Ee, 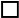), crypt abscess (Ca, 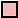), mucin depletion (Md, 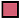) and immune cell infiltration (Ii, 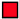) are indicated. Statistical analyses were conducted using a two-tailed paired *t*-test (**A**,**B**), two-way ANOVA with Sidak’s multiple comparisons (**C**), or one-way ANOVA with Tukey’s multiple comparisons (**D**,**E**). Each dot represents a quantitated result from one mouse. Red and green arrows point the presence of γH2AX in epithelial and immune cells respectively. DAI: disease activity index; error bars = SEM; HM: high magnification; *, *p* ≤ 0.05; ***, *p* ≤ 0.001; n.s., non-significant; a.u., arbitrary unit.

Perhaps most interestingly, γH2AX was detected in the DSS-induced inflamed colons, showing a clear dose-dependent increase by day 5, with maximal γH2AX^+^ cells at 3% DSS (Supplementary Figure S1D). Hematoxylin and eosin (H&E) staining revealed damage in the colitis tissues, such as the gradual loss of the epithelium layer, crypt structure and mucin (Supplementary Figure S1E, panels a–d). Immunostaining confirmed the γH2AX level (Supplementary Figure S1E, panels e–h). To refine the model, male mice were treated with 2.5% DSS for 7 days. This protocol reproduced key pathological features seen in both patients [46] and female mice, including weight loss (4% reduction, Figure 1A), significant colon shortening (27% reduction, Figure 1B), elevated disease activity index (DAI, Figure 1C) and a 13-fold increase in inflammation score (Figure 1D).

To mimic the pathological features of human UC, we established a chronic colitis mice model by administrating three cycles of 2.5% DSS for 7 days, each followed by a 2-week recovery, over a total of 70 days. This model exhibited an elevated inflammatory score, evaluated by the degree of epithelial erosion (Ee), crypt abscess (Ca), mucin depletion (Md), and immune cell infiltration (Ii) [47] (Figure 1D), alongside a corresponding increase in γH2AX accumulation (Figure 1E). Notably, tissue damage was less severe in the chronic colitis model than in the acute setting (Figure 1F, panels a–h), which is likely due to the recovery intervals. For example, epithelial erosion (Ee) was seldom found in the chronic colitis model (panels b and f compared to d and h). Accordingly, γH2AX staining was markedly reduced in the chronic colitis model compared to the acute colitis model (panels j and j’ compared to l and l’). Supporting this finding, the population (%) of γH2AX^+^ cells was also significantly raised in both acute and chronic DSS-treated male mice (Supplementary Figure S1F), indicating the presence of DSBs during inflammatory colitis.

### 3.2. Detection of γH2AX in Tissue Samples of Crohn’s Disease

**Figure 2:**
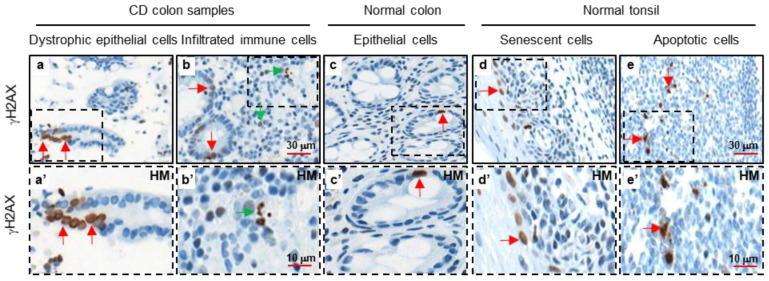
Detection of γH2AX in human Crohn’s disease. IHC images of γH2AX detection in CD colon samples (dystrophic epithelia cells, panels **a**, **a’**; infiltrated immune cells, panels **b**, **b’**), normal colon epithelial cells (panels **c**, **c’**) and normal tonsil (senescent cells, panels **d**, **d’**; apoptotic cells, panels **e**, **e’**) (three repetitions, *n* = 3). Red and green arrows indicate the presence of γH2AX in epithelial and immune cells respectively. HM: high magnification.

Similar to UC, patients with CD are at an increased risk of developing CRC. However, the pattern and severity of inflammation differ anatomically between these two diseases. In CD, tissue inflammation primarily locates in the colon and the terminal ileum, while patients with UC suffer inflammation largely in the distal colon and the rectum [3,45]. Notably, we also observed elevated levels of γH2AX in inflamed colon tissue samples of patients with CD. This accumulation was particularly evident in dystrophic epithelial (Figure 2, panel a; amplified image, panel a’) and immune cells within ulcerated regions of the CD colon samples (Figure 2, panel b; amplified image, panel b’) compared to normal colon epithelial cells (Figure 2, panel c; amplified image, panel c’). As a control, γH2AX staining was also detected in both senescent epithelial cells of the tonsil (Figure 2, panel d; amplified image, panel d’) and apoptotic cells located at the germinal centers of normal tonsil tissue (Figure 2, panel e; amplified image, panel e’). In summary, the above data support the notion that colonic tissues from CD patients exhibit significant γH2AX accumulation, consistent with DDR activation in the inflammatory state and potentially during cancer development [13,24,46].

### 3.3. TOP2 Inhibitors Antagonize Colitis-Associated γH2AX Expression in Mice

Having observed the accumulation of γH2AX during inflammatory colitis in both acute and chronic mouse models as well as in human CD samples, we next investigated the potential role of TOP2 isozymes in the generation of DSB and subsequent γH2AX signaling [18,47,48]. To assess the involvement of TOP2, we employed etoposide (Etop), which stabilizes the TOP2 cleavable complex (TOP2cc) by inhibiting the re-ligation step of the enzymatic cycle, thus promoting DSB formation. Differently, ICRF-187 (dexrazoxane) and ICRF-193 are catalytic inhibitors that actually exhibit weak TOP2cc-forming activity but have a great ability to antagonize/reduce TOP2-mediated DSB generation [49].

**Figure 3:**
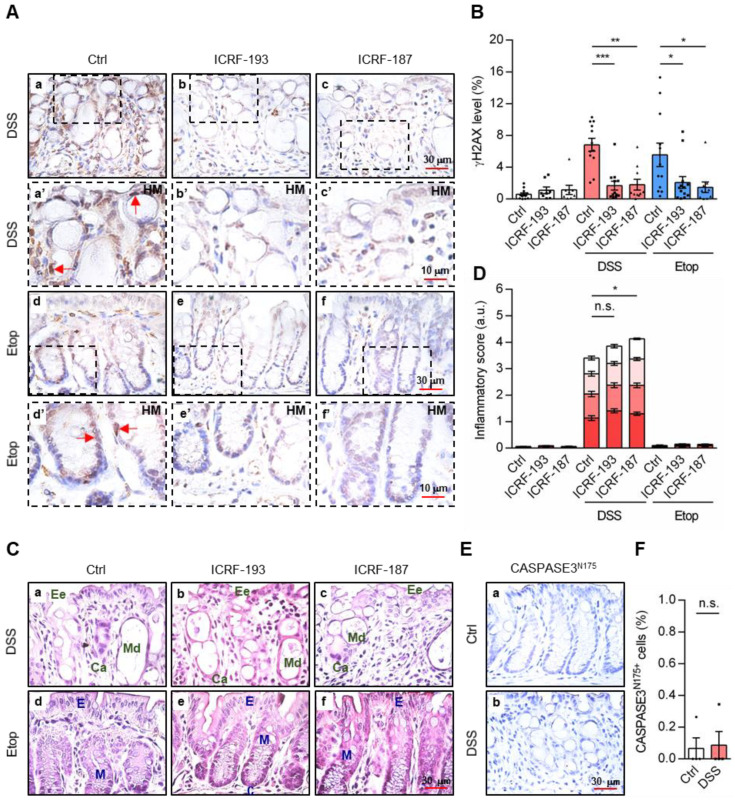
TOP2-antagonizing inhibitors reduced γH2AX accumulation. Mice were treated with TOP2-specific inhibitors ICRF-193 (2.5 mg/kg) and ICRF-187 (15 mg/kg). After 1 day, mice received either 7-day treatment of 2.5% DSS or Etop (40 mg/kg) (three repetitions, *n* ≥ 8). (**A**,**B**) Colon tissues were examined for the presence of γH2AX (panels **a**–**f**, and with high-magnification HM panels **a’**–**f’**) and quantified (DSS = 6.8 + 0.8, + ICRF-193 = 1.7 + 0.6, + ICRF-187 = 1.8 + 0.7%; Etop = 5.6 + 1.5, + ICRF-193 = 2.1 + 0.8, + ICRF-187 = 1.5 + 0.7%). The levels of γH2AX expression were measured and are shown as a percentage (%) of the area fraction. Red arrows indicate γH2AX expression in the epithelial cells. (**C**,**D**) Representative images of H&E staining (panels **a**–**f**) and quantitative analyses of the inflammatory score of mouse colon tissues. Pathologic structures of four inflammatory scoring parameters, including epithelial erosion (Ee, 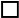), crypt abscess (Ca, 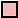), mucin depletion (Md, 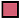) and immune cell infiltration (Ii, 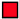) as well as normal epithelial (E) and mucin layers (M), were indicated and quantified. (**E**,**F**) Detection of apoptotic caspase 3N175+ in the colons of mice and quantitative results (one repetition, *n* = 4). Statistical analyses were conducted using a two-tailed paired *t*-test (E) and one-way ANOVA with Tukey’s multiple comparisons (**A**,**C**). Each dot represents a quantitated result from one mouse. Red arrows indicate the presence of γH2AX in epithelial cells. Error bars = SEM; *, *p* ≤ 0.05; **, *p* ≤ 0.01; ***, *p* ≤ 0.001; n.s., non-significant; HM: high magnification; a.u., arbitrary unit.

In the colon tissue of mice treated with 2.5% DSS, IHC using γH2AX antibodies revealed DSB accumulation, which was markedly reduced by co-treatments of ICRFs (visualized by brown marks; Figure 3A, panel a; amplified image, panel a’; quantitative results in Figure 3B). Notably, treatment of ICRF compounds alone only caused minimal γH2AX signals, thus serving as a control (Figure 3B). Consistently, both ICRF-193 or ICRF-187 significantly reduced γH2AX accumulation in Etop-treated mice (Figure 3A, panels d vs. e and f, magnified panels, d’ vs. e’ and f’; quantified in Figure 3B), where γH2AX detection primarily localized near the apical membrane of epithelial cells in Etop-treated colonic samples. We further validated the inhibitory effect of ICRFs by quantifying the percentage of γH2AX-positive cells in acute colitis and Etop-treated mice (Supplementary Figure S2A). Although ICRF caused modest weight losses (Supplementary Figure S2B) in both DSS- and Etop-treated mice, Etop and/or ICRF treatments did not modify colon length (Supplementary Figure S2C) and DAI score (Supplementary Figure S2D); ICRF treatments only enhanced the DSS-induced DAI activity. Importantly, neither Etop nor ICRFs treatments, alone or in combination, elicited a significant inflammatory response (Figure 3C). Etop did not induce any tissue damage (Figure 3C, panel d vs. a), and ICRF treatments only marginally affect DSS-stimulated inflammation (Figure 3C, panel b and c vs. a) and tissue damage (Figure 3D), despite their strong inhibition of Etop- and DSS-induced γH2AX accumulation (Figure 3B). Thus, the above results suggest that TOP2 might contribute to DSS-induced γH2AX activation.

### 3.4. Cellular DSS Treatment Induced DNA Breaks, Which Were Reduced by TOP2 Inhibitors

To strengthen the above notion, HCT116 human CRC cells were treated with DSS to induce γH2AX expression, and ICRFs significantly reduced DSS-induced γH2AX expression (Supplementary Figure S2E,F). To further validate that DSS induced DSBs, 53BP1 phosphorylation at S25/S29 (53BP1_pS25/S29_), another DSB marker, was examined [15]. Treatments of HCT116 CRC cells with either Etop or DSS not only caused γH2AX accumulation but also significantly upregulated the phosphorylation of 53BP1_pS25/S29_ (Supplementary Figure S3A,C; quantitative results in Figure S3B and Figure S3D, respectively). Notably, ICRF-187 and ICRF-193 significantly reduced both Etop- and DSS-induced 53BP1_pS25/S29_ levels (Supplementary Figure S3E; quantitative results in Figure S3F). Furthermore, it seems that γH2AX accumulation was not directly related to cell death since the acute DSS treatment did not induce apoptotic caspase activation (Figure 3E: quantitative results in Figure 3F). These findings suggest that DSB formation and γH2AX accumulation in response to DSS or Etop are primarily mediated by TOP2 activity rather than apoptotic cell death pathways.

### 3.5. TOP2-Antagonizing Inhibitors Reduce the Formation of Colon Adenoma

**Figure 4:**
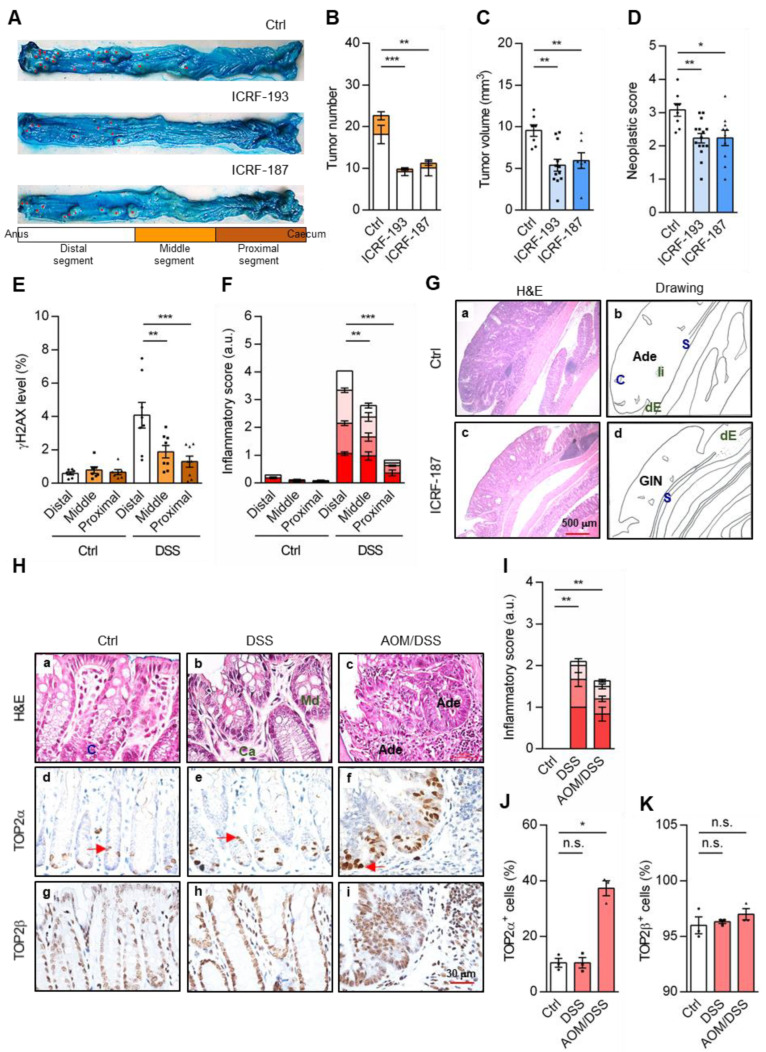
TOP2 inhibitors reduced colon cancer formation. The colon cancer disease model was built by administering mice with 10 mg/kg AOM followed by three cycles of 7-day 2.5% DSS treatment (AOM/DSS) (three repetitions, *n* ≥ 7). The mouse colons were opened longitudinally and stained with Alcian blue. Red dots indicate tumor locations. The distal position of the colon is closest to the anus. (**A**) The relative numbers of tumors in the distal (white), middle (light brown), and proximal (brown) segments were scored, and (**B**) quantitative results of the total numbers of tumors (distal = 18.1 ± 2.2, middle = 4.5 ± 1.0) and (**C**) tumor volume per colon, and (**D**) neoplastic score are shown. (**E**) Quantitation of relative intensities of γH2AX and (**F**) inflammatory signals at the three segments of the colon in an acute colitis setting (DSS) (two repetitions, *n* ≥ 7). (**G**) H&E staining of a tumor located in the distal colons of untreated (Ctrl; panel a, corresponding drawing in panel b) and ICRF-187-treated mice (panel c, drawing in panel d) (three repetitions, *n* ≥ 7). Adenoma (Ade, neoplastic score ≥ 3.0) and gastrointestinal neoplasia (GIN, neoplastic score ≥ 2.0) above the submucosa (S) layer, as well as features ranging from cribriform (sieve-like) structures (C), infiltrated immune cells (Ii), and dysplastic epithelium (dE), are indicated. (**H**) H&E and IHC images of mouse colonic tissues from chronic colitis (DSS) and cancer (AOM/DSS) disease models (H&E staining, panels a–c, and immunostaining of TOP2α, panels d–f, and TOP2β, panels g–i). Pathologic structures of 4 inflammatory scoring parameters, including crypt abscess (Ca, 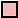), and mucin depletion (Md, 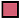), are indicated. Red arrows indicate the TOP2α in the epithelial cells. Quantitative analyses of (**I**) inflammatory score, (**J**) TOP2α^+^ and (**K**) TOP2β^+^ cells (one repetition, *n* = 3). Normal crypt structure (C) in the control tissues (Ctrl) and the tumor region (T) and the pathologic structures, including crypt abscess (Ca) and mucin depletion (Md), are indicated and were quantitated as described above. TOP2α^+^ and TOP2β^+^ cells were quantitated by manual cell counting (>100 cells per image, >2 images per sample; see Section 2). Statistical analyses were conducted using a two-tailed unpaired *t*-test (**B**–**D**), two-way ANOVA with Sidak’s multiple comparisons (**E**,**F**), or one-way ANOVA with Tukey’s multiple comparison (**I**–**K**). Each dot represents a quantitated result from one mouse. Error bars = SEM; *, *p* ≤ 0.05; **, *p* ≤ 0.01; ***, *p* ≤ 0.001; n.s., non-significant; a.u., arbitrary unit.

Having established a link between TOP2 activity and γH2AX accumulation in both acute and chronic colitis models, we next investigated whether TOP2-antagonizing inhibitors could prevent the development of colon cancer. To this end, mice were first treated with the tumor-promoting azoxymethane (AOM, first stage) and followed with three cycles of DSS treatment (second stage; total course 70 days; AOM/DSS cancer model) to initiate inflammation-associated colon adenoma [24,29,44]. After 70 days, mice treated with ICRF-193 and ICRF-187 exhibited a significant reduction in tumor burden, with an approximate 50% decrease in the average total number of tumors in the entire colon per mouse (Figure 4A, quantitative results in Figure 4B). These results suggest a role for TOP2 isozymes in promoting cellular proliferation and tumorigenesis. Indeed, by inhibiting both TOP2α and TOP2β isozymes, ICRF not only caused a decrease in cancer cell proliferation (i.e., tumor growth/volume; Figure 4C) but also slowed down tumor progression (i.e., neoplastic score; Figure 4D), supporting the potential of TOP2 inhibition as a strategy to suppress tumor progression in colitis-associated cancer.

### 3.6. Incidences of Proximal/Distal CRC Paralleled with γH2AX and Inflammation Levels

A spatial distribution of tumors on each colon was observed in the AOM/DSS-treated mice, with tumor formation preferentially occurring in the distal and middle segments but not in the proximal one (Figure 4A,B). Notably, this distribution pattern mirrored that of γH2AX accumulation (Figure 4E) and inflammatory score (Figure 4F), and both followed the same hierarchy: distal > middle > proximal. Consistently, γH2AX^+^ cells were also mainly detected in the distal segment, followed by the middle and proximal ones (Supplementary Figure S4A; quantitative results in Figure S4B). AOM/DSS treatment only slightly activated apoptotic caspase 3^N175^ (Supplementary Figure S4C; quantitative results in Figure S4D). Collectively, these results suggest that inflammation-associated γH2AX accumulation correlates with tumor formation in a segment-specific manner along the colon. Consistent with the tumor images represented in Figure 4G and Supplementary Figure S4E, the majority of the tumors in the AOM/DSS model progressed to gastrointestinal neoplasia (GIN, neoplastic score ≥ 2.0, Figure 4D). These tumor lessons typically displayed up-growth of tubular and/or villous neoplasm arising from the dysplastic epithelium (dE). In the control group (Ctrl, AOM/DSS-treated), many tumors reached the stage of adenomas (Ade; neoplastic score ≥ 3.0, Figure 4D), with a higher nucleus/cytoplasmic ratio, a marked reduction in inter-glandular stroma, an increase in space with a cribriform (sieve-like) structure (C) and prominent infiltrated immune cells (Ii).

Immune cell clusters were detected in the basal epithelial layer near and within the submucosa (S). Further histological analysis revealed that tumors in the colons of the AOM/DSS-treated mice (Ctrl, Figure 4G and Supplementary Figure S4E, panels a,b) were larger and at a more advanced stage compared to those in the ICRF-treated groups (panels c,d). Notably, ICRF-193 effectively inhibited cancer progression from gastrointestinal neoplasia to adenoma (Supplementary Figure S4E). Consistent with this reduced tumor progression, neoplastic lesions in the ICRF-187-treated mice exhibited a lower nuclear-to-cytoplasmic (N/C) ratio and fewer cribriform glandular structures compared to controls (Figure 4G, panel c vs. a), reflecting less aggressive tumor histopathology.

### 3.7. TOP2 Isozymes Expression During Tumorigenesis

Considering that TOP2α and TOP2β exhibit similar sensitivities to ICRFs but with different physiological and/or pathological roles [19], we explored whether these two TOP2 isozymes might contribute differentially to the tumorigenic process. Both chronic DSS colitis and AOM/DSS tumor models displayed similar decreases in body weight and colon length over the course of disease progression (Supplementary Figure S4F,G). In addition, H&E staining revealed that the tumors exhibited more compacted and disorganized structures (Figure 4H, panels c compared to a and b), characterized by a notable loss of crypt structures (C) in the adenoma region (Ade, panel c). Both models visually induced inflammatory responses compared to the control (Figure 4H, upper panels a–c; quantitative results in Figure 4I).

To investigate the link between tumorigenesis and TOP2 isozyme expression, we performed IHC staining. TOP2α expression was markedly elevated in the adenoma tissues, as shown by the increase in TOP2α^+^ cells (Figure 4H, panels d vs. f; quantitative results in Figure 4J), which is consistent with the proliferative function of TOP2α [49,50]. In parallel, we observed a 4-fold increase in cells detected with the proliferation marker Ki-67^+^ in tumor regions (Supplementary Figure S4H, panels a–c; quantitative results in Figure S4I). On the contrary, TOP2β expression (represented by TOP2β^+^ cells) remained relatively unchanged across chronic inflammatory and tumorigenic processes (Figure 4H, panels g–i; quantitative results in Figure 4K). These above data suggested that the level of expression of each TOP2 likely implies their distinct roles in different cell cycle stages [47,48,50] and initiation of tumorigenesis.

### 3.8. TOP2β-Mediated DSB and Tumorigenesis

**Figure 5:**
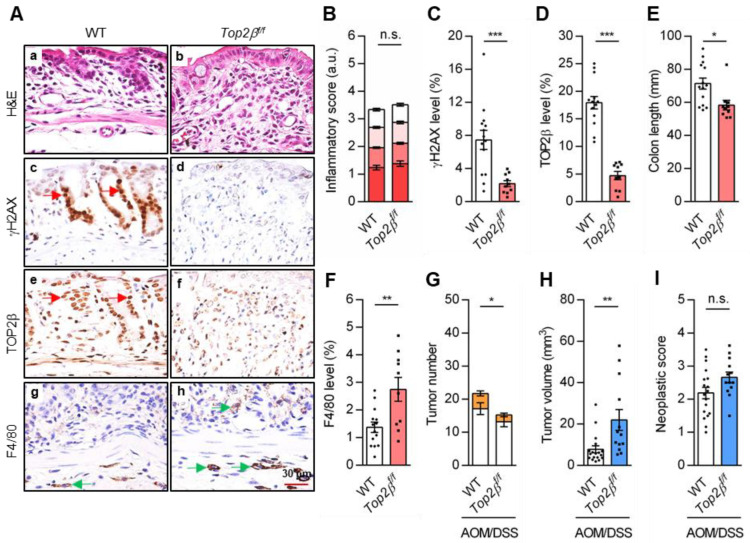
The *Top2β^f/f^* mice had reduced colitis-associated γH2AX accumulation and tumorigenesis. Mice with acute colitis and cancer disease models were established as described in the Materials and Methods. (**A**) Images of mouse colonic tissues: H&E staining (panels a,b) and IHC immunostaining for γH2AX (panels c,d), TOP2β (panels e,f), and F4/80 (panels g,h). Red arrows point to the presence of γH2AX and TOP2β in the epithelial cells, while green arrows indicate F4/80 in infiltrated macrophages. Quantitative results of (**B**) inflammatory score, (**C**) γH2AX accumulation (WT = 7.5 ± 1.1, *Top2β^f/f^* = 2.2 ± 0.4), (**D**) TOP2β expression, (**E**) colon length and (**F**) F4/80 staining (four repetitions, *n* ≥ 10). (**G**) Quantitative analyses of total tumor number and (**H**) volume per colon, and (**I**) neoplastic score in the AOM/DSS cancer setting (five repetitions, *n* ≥ 13). Statistical analyses were conducted using a two-tailed unpaired *t*-test (**G**–**I**). (**I**, *p* = 0.053, n.s.). Each dot represents a quantitated result from one mouse. Error bars = SEM, *, *p* ≤ 0.05, **, *p* ≤ 0.01, ***, *p* ≤ 0.001; n.s., non-significant; a.u., arbitrary unit.

To further explore the role of TOP2β in DSB generation, we employed the *Top2β* conditional knocked-out mouse strain generated via the villin-Cre-loxP recombination mechanism (genotyping, Supplementary Figure S5A,B). In the 7-day acute colitis model, wild-type (WT) and *Top2β*-deficient (*Vil-Cre Top2β^flox2/flox2^*, *Top2β^f/f^*) mice displayed similar levels of inflammation (Figure 5A, panels a,b; quantitative results in Figure 5B). However, γH2AX accumulation and the population of γH2AX^+^ cells were greatly reduced by up to 70% in the colon tissues of the *Top2β^f/f^* mice (Figure 5A, panels c,d, quantitative results in Figure 5C; Supplementary Figure S5C). IHC showed a comparable 70% reduction in TOP2β protein expression in the colons of *Top2β^f/f^* mice compared to that of WT mice (Figure 5A, panels e,f; quantitative results in Figure 5D). Although *Top2β^f/f^*-deficient mice exhibited a slightly shorter colon (Figure 5E) and a slight increase in DAI activity (Supplementary Figure S5D) during acute colitis, there was no significant impact on body weight (Supplementary Figure S5E). Interestingly, these mice also showed elevated macrophage infiltration, as detected by increased F4/80 staining (Figure 5A, panels g,h; quantitative results in Figure 5F), a known feature of inflammatory colitis in both mice and UC patients [51]. To further validate TOP2β in DSS-activated γH2AX expression, the lentivirus-mediated TOP2β knockdown of the shTop2β and control shLuc HCT116 cell lines were treated with DSS or Etop to induce γH2AX expression (Supplementary Figure S5F,G). Consistently, both levels of DSS- and Etop-induced γH2AX activation in the shTop2β HCT116 cells were significantly lower. Together, our cellular and animal results strongly suggest a role of TOP2β in DSB generation during colitis.

The *Top2β^f/f^* mice had up to 30% fewer tumors in their proximal and middle colon segments (Figure 5G and Supplementary Figure S5H). In the *Top2β^f/f^* mice, the tumor volume was significantly larger (Figure 5H), likely due to the compensatory increased expression of TOP2α (Supplementary Figure S5I; 3.6-fold). Moreover, like the tumors developed in the WT mice (Figure 4H,J; 3.5-fold higher in cancerous samples), TOP2α expression also increased in the tumor tissues of the AOM/DSS *Top2β^f/f^* mice (Supplementary Figure S5I,J). Despite the increased TOP2α expression, there was a reduction in γH2AX accumulation (3.5-fold) in the *Top2β^f/f^* mice (Figure 5C), while the neoplastic scores remained similar (Figure 5I). Thus, our data underscore the potential role of TOP2β in DSB formation and cancer initiation, whereas TOP2α might play a more prominent role in tumor progression.

### 3.9. NO Stimulated TOP2β-Mediated DNA Breaks

**Figure 6:**
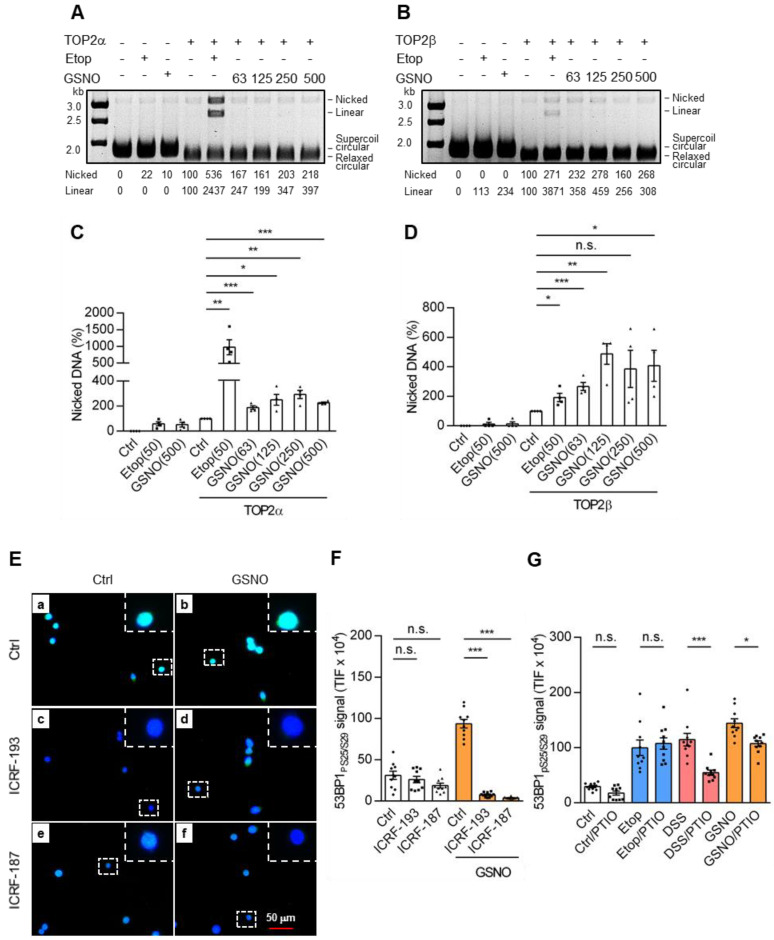
NO stimulated TOP2 isozymes to generate DNA breaks. (**A**,**B**) Circular plasmid DNA cleavage assays using recombinant TOP2α and TOP2β with increasing amounts of GSNO (63–500 μM) and Etop (50 μM), as indicated at the top of each panel. (**C**,**D**) Quantification of isozyme-generated SSBs (nicked DNA; **C**, by TOP2α, **D**, by TOP2β; four repetitions, *n* = 4). (**E**) Detection of 53BP1_pS25/S29_ in HCT116 cells grown on cover slips, further exposed to GSNO (100 μM, 4 h) with or without ICRF-187 and ICRF-193, and then subjected to IF analysis (400× image; 1000×, dashed square of the magnified inset), as described in the Materials and Methods. (**F**) The quantification data were from at least 5 images with 10 cells per group (three repetitions, *n* = 10). (**G**) HCT116 cells were treated with PTIO (100 μM, 4 h), Etop (50 μM, 1 h), DSS (5.0%, 4 h), and GSNO (100 μM, 4 h) as well as their combinations, as indicated. For the NO scavenger, cells were co-treated with PTIO and either Etop (1 h) or DSS (4 h) and then subjected to immunofluorescence analysis and quantitated (three repetitions, *n* = 10). Statistical analyses were conducted using a two-tailed unpaired *t*-test (**C**,**D**) or one-way ANOVA with Tukey’s multiple comparisons (**F**,**G**). Each dot represents a quantitated result from one cleavage reaction (**C**,**D**) or one cell (**F**,**G**). TIF: total immunofluorescence; kb: kilobase; *, *p* ≤ 0.05; **, *p* ≤ 0.01; ***, *p* ≤ 0.001; n.s., non-significant.

We investigated whether nitric oxide (NO), generated by nitric oxide synthase 2 (NOS2), which plays a pathogenic role during inflammation, could mediate DNA break formation. An in vitro cleavage assay was first set up using purified TOP2α and TOP2β recombinant proteins (Supplementary Figure S6A) as well as *S*-nitroso-glutathione (GSNO) as a source of bioavailable NO and circular plasmid DNA as a substrate (Supplementary Figure S6B). Both TOP2α and TOP2β completely relaxed the supercoiled pRYG at a molar ratio of 2. The corresponding DNA nicks and DSBs from each TOP2 isozyme alone were used as a 100% value of DNA cleavage. Under these experimental conditions, GSNO dose-responsive changes in the levels of nicked (SSBs) and linearized DNA (DSBs) by TOP2α (Figure 6A) or TOP2β (Figure 6B) were observed. Quantitation analyses showed that TOP2β was more sensitive to GSNO in generating SSBs than TOP2α (Figure 6C,D). Similar to SSB generation, the highest levels of DSBs (i.e., linear DNA) were obtained at GSNO concentrations of 250 μM with TOP2α (193 ± 33%) and 125 μM with TOP2β (671 ± 341%).

Similar results were obtained with linearized plasmid DNA substrates (Supplementary Figure S6C). For example, the formation of DNA breaks by TOP2β was 2-fold higher than that by TOP2α at a GSNO concentration of 31.3 μM. GSNO itself did not cause any DNA breakage (Supplementary Figure S6D). Of note is the fact that addition of EDTA decreased/reversed the Etop- and GSNO-induced DNA breaks mediated by both TOP2α (Supplementary Figure S6E,F) and TOP2β (Supplementary Figure S6G,H).

To further support the contributions of NO and TOP2 to the generation of DSBs, HCT116 cells were exposed to GSNO. As shown by IF and WB analyses, GSNO increased γH2AX and 53BP1_pS25/S29_ signals, whose expressions were reduced upon additional treatment of ICRFs (Figure 6E, compare panels c and e with d and f; quantitative results in Figure 6F; Supplementary Figure S7A, quantitative results in Figure S7B). Co-treatment of these cells with the NO scavenger carboxyl-PTIO (PTIO) also significantly reduced DSS- and GSNO-stimulated γH2AX (Supplementary Figure S7C, quantitative results in Figure S7D) and 53BP1_pS25/S29_ (Supplementary Figure S7E, compare panels a’ to b’ for DSS and c’ to d’ for GSNO; quantitative results in Figure 6G), while it did not influence the level of Etop-induced γH2AX and 53BP1_pS25/S29_ (Supplementary Figure S7C,D; Supplementary Figure S7E, compare panels g’ to h’). Collectively, our in vitro and in vivo data support the role of NO in regulating TOP2 activity for the formation of DNA breaks.

### 3.10. NOS2 Expression and NO Contribute to Colitis DNA Damage and Tumorigenesis

**Figure 7:**
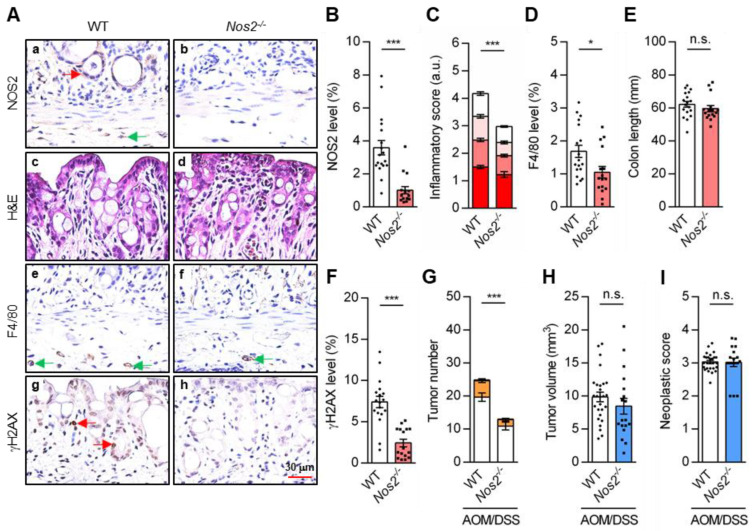
The pathological features of *Nos2^-/-^* mice. (**A**) Colon tissue samples from WT and *Nos2^-/-^* mice in an acute colitis (DSS) setting were subjected to H&E (panels c,d) and IHC analyses (NOS2 panels a,b, F4/80 panels e,f, γH2AX panels g,h). Red and green arrows indicate the presence of γH2AX in epithelial and immune cells respectively. Quantitation of (**B**) NOS2 expression, (**C**) inflammatory score, (**D**) F4/80 signal, (**E**) colon length and (**F**) γH2AX accumulation (four repetitions, *n* ≥ 16). Colon tissue samples from WT and *Nos2^-/-^* mice in cancer (AOM/DSS) setting were investigated. Quantitation of (**G**) total numbers of tumors and (**H**) tumor volume per colon, and (**I**) neoplastic score in cancer progression model (four repetitions, *n* ≥ 18). Statistical analyses were conducted using a two-tailed unpaired *t*-test (**B**–**I**). Each dot represents a quantitated result from one mouse. Error bars = SEM; *, *p* ≤ 0.05; ***, *p* ≤ 0.001; n.s., non-significant.

Activated macrophages with NOS2 expression, an inflammatory response mediated by the Toll-like receptor 4 (TLR4) pathway, infiltrated into inflamed tissues during colitis [5,16,51]. Experiments showed that Raw264.7 macrophage-like cells treated with lipopolysaccharide (LPS) and interferon γ (IFNγ; L/I) resulted in a high expression of NOS2 and NO production at 8 h (Supplementary Figure S8A,B). To further investigate the contribution of NO to tumorigenesis, an acute colitis disease model was established using Nos2-defective (*Nos2^-/-^*) mice (genotyping, Supplementary Figure S8C,D), resulting in decreases in NOS2 protein expression (Figure 7A, panels a,b; quantitative data in Figure 7B) and NO production [32]. The loss of NOS2 in (*Nos2^-/-^*) mice exhibited lower degrees of tissue damage (Figure 7A, panels c,d), inflammation (Figure 7C) and DAI scores (Supplementary Figure S8E) induced by the DSS treatment, but it did not result in a significant impact on body weight (Supplementary Figure S8F). The macrophage infiltration measured on the 7th day after treatment was reduced compared to the DSS-treated WT mice (Figure 7A, panels e,f; quantitative data in Figure 7D), which is in contrast to the increase in the *Top2β^flf^* mice (Figure 5F). Colon lengths of the WT and *Nos2^-/-^* mice were similarly shortened (Figure 7E).

Acute colitis-induced γH2AX detection reduced greatly in the *Nos2^-^*^/-^ mouse colons compared to the WT mice (Figure 7A, panel g,h; quantitative results in Figure 7F and Supplementary Figure S8G). This γH2AX decrease is correlated with the reduction in NOS2 protein (Figure 7B). To understand the involvement of NOS2 in tumor formation, WT and *Nos2^-/-^* mice were treated with AOM and DSS to induce tumors. There was a significant reduction in total tumor number in *Nos2^-/-^* mice (Figure 7G and Supplementary Figure S8H), whereas tumor volume and neoplastic score (in the adenoma stage) were similar (Figure 7H,I and representative image in Supplementary Figure S8I).

### 3.11. TOP2 Isozymes and NOS2 Partner in γH2AX Activation and Cancer Initiation

To further understand the functional relationship between TOP2 isozymes and NOS2 (which resulted in γH2AX and 53BP1_pS25/S29_ accumulation and subsequently led to colon cancer initiation), two sets of experiments were performed in the *Nos2^-/-^* mice using either a pharmacological (with a TOP2α/2β inhibitor ICRF-193) or a genetic (with the Cre/loxP technology on TOP2β) approach.

**Figure 8:**
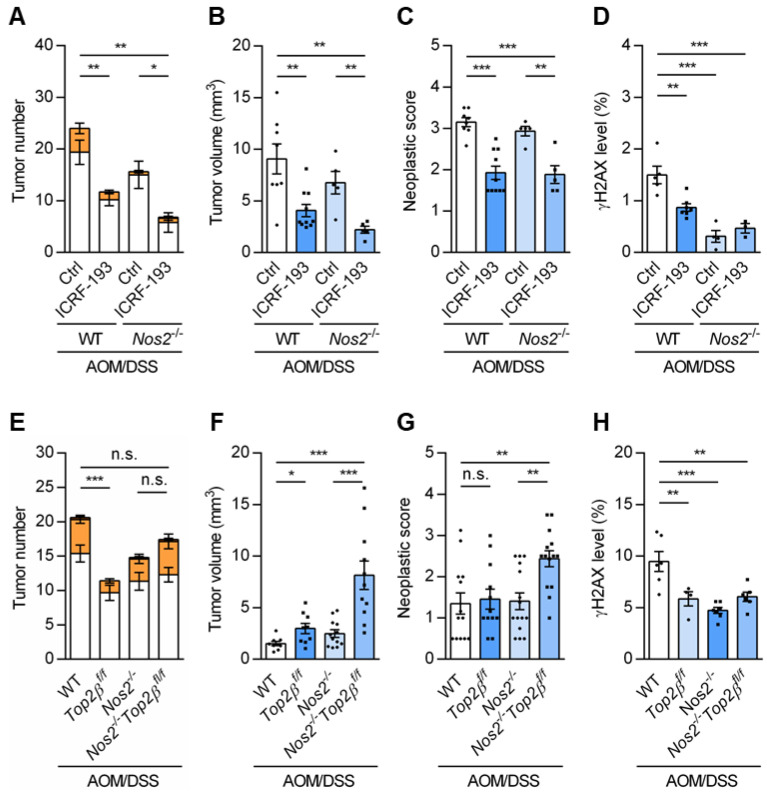
Contribution of TOP2 isozymes and NOS2 to cancer development. Quantitation of (**A**) total tumor number per colon, (**B**) tumor volume, and (**C**) neoplastic score and (**D**) γH2AX accumulation in ICRF-193 treatment of WT and *Nos2^-/-^* mice in the AOM/DSS cancer setting. The AOM/DSS cancer disease model (with or without 10 mg/kg ICRF-193) was performed in WT and *Nos2^-/-^* mice, as described in Figure 4 (10 mg/kg AOM followed by three cycles of 7-day 2.5% DSS; total of 70 days; two repetitions, *n* ≥ 3). Lower-intensity treatments of AOM (10 mg/kg) and DSS (2.0%, 5 days; three cycles) also resulted in colon cancer formation in the WT, *Top2β^f/f^*, *Nos2^-/-^* and *Nos2^-/-^ Top2β^f/f^* mice (total of 77 days). Quantitation of (**E**) total tumor number per colon, (**F**) tumor volume, (**G**) and neoplastic score and (**H**) γH2AX accumulation were determined on day 77 (five repetitions, *n* ≥ 4). Statistical analyses were conducted using a two-tailed unpaired *t*-test (**A**–**C**, **E**–**G**) and one-way ANOVA with Tukey’s multiple comparison (**D**,**H**). Each dot represents a quantitated result from one mouse. Error bars = SEM, *, *p* ≤ 0.05, **, *p* ≤ 0.01, ***, *p* ≤ 0.001; n.s., non-significant.

Pharmacologically, treatments of the WT and *Nos2^-/-^* mice with ICRF-193 reduced the total tumor number per colon (Figure 8A), the tumor volume (Figure 8B) and the neoplastic score (Figure 8C). From anatomical and pathological points of view, ICRF-193 significantly arrested cancer initiation and progression/evolution from mild neoplasia with aberrant crypt foci (ACF) and/or GIN into low-grade adenoma (Figure 8C and Supplementary Figure S8J), thus underlying a partnership between TOP2 isozymes and NOS2. Interestingly, such tumor formation (evaluated by tumor number, Figure 8A) seems to parallel the γH2AX levels accumulated in mouse colons of different treatment groups (Figure 8D).

When using a genetic approach, we generated a *Top2β^f/f^* knock-in deficiency in WT or *Nos2^-/-^* mouse colons. Notably, *Nos2^-/-^ Top2β^f/f^* double-knockout mice showed increased susceptibility, necessitating a reduction in the DSS treatment from 2.5% to 2.0% over an extended period (11 weeks) in the AOM/DSS cancer model. Under these conditions, tumor propensities in WT mice were less apparent. On day 77, *Top2β^f/f^*, *Nos2^-/-^* and *Nos2^-/-^ Top2β^f/f^* mice exhibited fewer tumors per colon compared to WT mice (Figure 8E). However, *Top2β^f/f^* deficiency significantly promoted tumor growth in both WT and *Nos2^-/-^* mice (Figure 8F). The neoplastic score of the *Nos2^-/-^ Top2β ^f/f^* mice was higher than that of the *Nos2^-/-^* mice (Figure 8G, images in Supplementary Figure S8K), despite their lower reproduction rate. Additionally, γH2AX accumulation was reduced across all three mutant models (Figure 8H), suggesting that TOP2β and NOS2 might contribute to γH2AX accumulation, thus influencing cancer initiation. Considering the observed TOP2α upregulation in the *Top2β^f/f^*-deficient mice (Supplementary Figure S5G) and pharmacological inhibition data (Figure 8A–D), our genetic analysis potentially underscores a primary role for TOP2β in tumor formation, whereas TOP2α appears to be more involved in cancer progression.

## 4. Discussion

Murine tumor models with both male and female mice serve as valuable tools for recapitulating human cancer and are particularly useful in preclinical studies of intestinal cancer. IBD, including UC and CD, primarily affect the colon and might initiate and further progress along the adenoma–carcinoma sequence, ultimately contributing to CRC development [7,52,53,54,55,56]. Our study focused on documenting early stages of cancer development through in vivo analyses of colon tissue, assessing DNA breaks and inflammatory and oncogenic biomarkers. We sequentially established several mouse models that mimic IBD-like colon inflammation (i.e., DSS-UC mouse models, including acute and chronic colitis) as well as inflammation-associated cancer (i.e., AOM/DSS CRC model), and we tracked the evolution of clinical and pathological features over a long period of time. Importantly, male and female mice exhibited similar pathological features. Using a combination of pharmacological and genetic approaches, we examined key pathological parameters, including inflammation, DAI, colon length, tumor number, volume and neoplastic progressing, and we correlated these with the presence of DNA breaks and the involvement of TOP2s and NOS2. Coupled with reports connecting TOP2, NOS2 and inflammation to DNA damage responses [11,17,25,56,57], our study thus discussed a key role of TOP2β and NOS2 in CRC tumorigenesis associated with colitis-induced DNA breaks.

In this regard, our DSS-UC and AOM/DSS CRC models recapitulated pathological hallmarks of human UC and aspects of CD. Inflammation was induced along the large intestine, thus facilitating dissemination of pro-inflammatory intestinal contents and subsequent cancer development. During the course of these diseases, histological analyses revealed pathological characteristics of human colitis, including mucin depletion, crypt abscess, epithelial erosion and immune cell infiltration, further validating the clinical relevance of these models in studying the link between inflammation, DNA lesions, and early tumorigenesis [11,26,58,59].

We specifically identified DNA breaks in colon tissue samples, as revealed by the presence of γH2AX and 53BP1_pS25/S29_ markers: the former indicates the phosphorylation of H2AX by PI3-family kinases (including ATM, ATR and DNA-PK) [60], while the latter is recruited at DSB sites and is indicative of an active repair process (likely via the NHEJ pathway) [15]. Importantly, our results further suggest that DNA breaks in colitis might be driven by TOP2 and NOS2. Several key observations support this notion: (i) γH2AX accumulations in both acute and chronic colitis are reduced by TOP2-anatgonizing ICRF-193 and ICRF-187; (ii) both DSS-caused γH2AX and 53BP1_pS25/S29_ increases were attenuated upon administration of either ICRF-193, ICRF-187 or the NO scavenger PTIO; and (iii) silencing TOP2β and NOS2 (in the *Top2β^f/f^* and *Nos2^-/-^* mice respectively) led to marked reduction in DSB detection in colon tissues. Despite some limitations, these findings collectively highlight a leading role of both TOP2 and NO in generating DSBs and promoting tumorigenesis. This conclusion is further strengthened when observing how the knockout/loss of either NOS2 or TOP2β in colon tissues not only significantly reduced γH2AX levels but also parallelly lowered the tumor burden in the *Nos2^-/-^* or *Top2β^f/f^* mice.

NOS2 expression has been reported to be elevated during inflammation [51], and our results showed that NOS2 expression resulted in NO production. These NO molecules then stimulated TOP2 activity and formation of DNA breaks. Such DSS-induced and TOP2-mediated DNA breaks (noted upon accumulation of γH2AX and 53BP1_pS25/S29_) are also in line with our previous study [17] reporting that when cancer cells were exposed to GSNO, the level of DSB was reduced upon treatment with PTIO. Complementing the above observations in vivo and in cells, our in vitro experiments showed that NO stimulates both recombinant TOP2 isozymes to introduce breaks in plasmid DNA. We thus argue that, during colitis, stimulated NOS2 expression results in production of NO, which then causes proper TOP2 nitrosylation, possibly at the thiol group(s) of cysteine(s), subsequently leading to formation of TOP2cc, DNA break(s) and, later, γH2AX accumulation. Further TOP2 modifications at higher NO concentration(s) inactivate the enzymatic activity, which is also consistent with the concentration in our in vitro cleavage results. Future studies would be needed to identify the nitrosylation sites on TOP2 isozymes and to dissect the molecular mechanism by which NO regulates TOP2 activity to promote DNA breaks. In addition to representing DSBs, γH2AX accumulation might also be considered as involving non-classical mechanisms, such as apoptosis, replication fork arrest or prolong single-stranded breaks (SSBs). The earlier detection of γH2AX, however, does not match with the three non-classical events above, which require lengthy processes to activate γH2AX. In agreement, our results showed that apoptotic markers did not correlate with γH2AX accumulation.

Our observations on the formation of DNA breaks and the chromatin modification might also suggest that DNA processing activities were activated, thus raising questions regarding the efficiency of the NHEJ mechanism in repairing such damage. In this regard, proteasome activity has been reported to modulate the activation of repair pathways for TOP2-induced DNA breaks, such as NHEJ (by DNA-PK phosphorylation). Moreover, the observed increase in TOP2 activity might also reflect an elevated transcriptional state (namely transcriptional addiction) of cancer cells [61,62,63]. Supportively, several studies have shown that TOP2β, either alone or in combination with repair enzymes DNA-PK and PARP1, is recruited to promoters and regulates chromatin organization [20,49,50,64]. Notably, at the activated gene promoters, these proteins could induce transient DNA breaks, thus further coordinating accessibility and allowing for accurate gene expression by RNA polymerase [20,49,64,65,66]. However, if such DNA breaks are not properly repaired, their accumulation may promote genomic instability, including gene mutations and chromosomal translocations, thus accelerating tumorigenesis and cancer development. Further study using advanced technologies such as single-cell RNA-seq analysis might offer deeper understanding of this molecular pathology.

In our mouse models, we noticed a consistent pattern, wherein the γH2AX level, inflammatory degree and tumor incidence were more predominant at the distal segment of the mouse colon. It is worth noting that γH2AX was found localized to some cells, likely those undergoing replication. This preferential tumor localization, inflammation response and γH2AX detection closely resembles what was observed in the distal segment of the human CRC subtype, characterized by CIN and oncogenic translocations [56]. Since we observed no significant change in inflammatory score in the DSS-treated *Top2β^f/f^* deficient mice, the higher distal expression of TLR4 (a key receptor to activate inflammatory responses) [51] might thus offer a plausible explanation for elevated inflammation at the distal colon segment, thereby facilitating DNA damage and tumor formation.

Our results also argue that TOP2 isozymes contributed to different stages of cancer development (in cancer initiation for TOP2β and tumor growth for TOP2α) based on the following: (i) inhibition of both TOP2α and TOP2β by ICRFs in the DSS-treated mice reduced γH2AX detection but had only a minimal effect on tissue damage and overall inflammation in the colons; (ii) both γH2AX accumulation and tumor formation were markedly reduced in the colons of the *Top2β^f/f^* (despite the 4-fold increase in TOP2α expression) and ICRF-193-treated mice; and (iii) ICRF-193 treatment (which inhibits TOP2α) reduced tumor growth, while *Top2β^f/f^* deficiency (in which TOP2α is elevated) decreased tumor burden but increased tumor growth in cancer mouse models. Our results suggest that the induction of DNA breaks by TOP2 enzymes is potentially mediated through nitrosylation by NOS2-produced NO during colitis. Cysteine nitrosylation on proteins is one of the important biological impacts of NO, while TOP2 thiol modifications induce TOP2cc-meidated DNA breaks and subsequent γH2AX activation. Thus, NOS2 expression in colitis leads to NO production with subsequent TOP2cc formation and γH2AX accumulation.

It should be noted that TOP2-mediated DNA breaks are typically short-lived and highly reversible under physiological conditions, but their persistence during colitis may contribute to tumorigenesis. We previously reported that RNAi-mediated knockdown (KD) of each TOP2 isozyme in either HCT116 human colorectal carcinoma cells or HL-60 human leukemia cells resulted in differential degrees of DNA breaks induced by etoposide or a NO donor, GSNO [17]. These KD experiments demonstrated that TOP2β predominantly mediates DNA damage and genome instability upon NO exposure, while TOP2α is more responsive to etoposide-induced DNA cleavage and cancer cell death. This functional preference aligns with our current findings that suggest the potential of TOP2β primarily in tumor formation, whereas TOP2α might be involved in tumor growth and progression. Supporting this conclusion, TOP2α expression in tumors is elevated, and TOP2α has been identified as a major determinant of cancer cell sensitivity to TOP2-targeting drugs (e.g., doxorubicin) [19,67].

## 5. Conclusions

Using mouse acute, chronic colitis and cancer disease models, our results mimic how colonic inflammation in UC and CD patients might lead to the formation of DNA breaks, which could then subsequently lead to tumorigenesis. We further show that such DNA breaks are largely the result of TOP2 activities, which are, in turn, regulated by elevated NOS2 expression with higher NO production in inflamed colon tissues. Inhibition or silencing of either TOP2 or NOS2 significantly reduces DSB accumulation and results in a consequent decrease in tumorigenic initiation and cancer progression. This present work not only provides a better understanding of the pathogenic mechanisms for initiation and development of colitis-associated cancer [68,69,70] but also helps to identify precision intervention strategies targeting the TOP2-NOS2 axis for cancer prevention and treatment.

## Data Availability

All the data are available from the corresponding author upon request.

## Supplementary Materials

The following supporting information can be downloaded at https://www.mdpi.com/article/10.3390/cancers18101519/s1: We include eight Supplementary Figures, seven Supplementary Tables, one Supplementary Method, and five Supplementary References. Supplementary Figure S1 DNA damage responses and pathological features of mice after DSS treatments, and thus highlighting a similar dosage-dependent induction of DNA damage responses after DSS treatment. Supplementary Figure S2 TOP2-antagonizing inhibitors on the DSS-UC or etoposide-treated mice, and thus confirming the impact of TOP2-antagonizing ICRF-187 and ICRF-193 inhibitors in the colons of the DSS- or etoposide-treated mice. Supplementary Figure S3 53BP1 phosphorylation under etoposide and DSS treatments, and thus further showing changes in 53BP1 phosphorylation at Ser25/29 under etoposide and DSS treatments. Supplementary Figure S4 The effect of ICRF-193 on tumor progression in the colon cancer disease model, and therefore revealing the effect of ICRF-193 on tumor progression in the colon cancer disease model. Supplementary Figure S5 Generating a colon adenoma disease model with the *vil-Cre Top2β^f/f^* colonic conditional knockout mice, and thereby describing the generation of a colon adenoma disease model with the *vil-Cre Top2β^f/f^* colonic conditional knockout mice. Supplementary Figure S6 Nitric oxide activated TOP2\α- and TOP2β- mediated DNA breaks. Supplementary Figure S7 Both DSS and GSNO treatments induced γH2AX and 53BP1_pS25/S29_ detections, and thus demonstrating that DSS and GSNO treatment induced NO-activated 53BP1_pS25/S29_. Supplementary Figure S8 Nos2 deficiency caused a reduction in DAI score, but not in body-weight and tumor growth/progression, and thus focusing on the *Nos2* deficiency causing a reduction in DAI score but not in body weight and tumor growth/progression. Supplementary Table S1. Primer sequences for mouse genotyping. Supplementary Table S2. The shRNA Sequences for Lentivirus Knockdown of Luciferase and TOP2β. Supplementary Table S3. Antibodies. Supplementary Table S4. Special chemicals and reagents. Supplementary Table S5. Bacteria, yeast and cell growth media. Supplementary Table S6. Critical commercial assays. Supplementary Table S7. Cell lines, bacteria, yeast, Lentiviruses, mouse strains.

## References

[1] Jess T., Rungoe C., Peyrin–Biroulet L. (2016). Risk of Colorectal Cancer in Patients with Ulcerative Colitis: A Meta-analysis of Population-based Cohort Studies. Clin. Gastroenterol. Hepatol..

[2] Dyson J.K., Rutter M.D. (2012). Colorectal Cancer in Inflammatory Bowel Disease: What is the Real Magnitude of the Risk?. World J. Gastroenterol..

[3] Santos J.C.D., Malaguti C., Lucca F.A., Cabalzar A.L., Ribeiro T., Gaburri P.D., Chebli L.A., Chebli J.M.F. (2017). Impact of Biological Therapy on Body Composition of Patients with Chron’s Disease. Rev. Assoc. Med. Bras. (1992).

[4] Keller D., Windsor A., Cohen R., Chand M. (2019). Colorectal Cancer in Inflammatory Bowel Disease: Review of the Evidence. Tech. Coloproctol..

[5] Hanahan D., Weinberg R.A. (2011). Hallmarks of Cancer: The Next Generation. Cell.

[6] Li J., Ma X., Chakravarti D., Shalapour S., DePinho R.A. (2021). Genetic and Biological Hallmarks of Colorectal Cancer. Genes Dev..

[7] Shah S.C., Khalili H., Gower-Rousseau C., Olen O., Benchimol E.I., Lynge E., Nielsen K.R., Brassard P., Vutcovici M., Bitton A. (2018). Sex-Based Differences in Incidence of Inflammatory Bowel Diseases-Pooled Analysis of Population-Based Studies From Western Countries. Gastroenterology.

[8] Sanchez-Vega F., Mina M., Armenia J., Chatila W.K., Luna A., La K.C., Dimitriadoy S., Liu D.L., Kantheti H.S., Saghafinia S. (2018). Oncogenic Signaling Pathways in the Cancer Genome Atlas. Cell.

[9] Gómez-Herreros F. (2019). DNA Double Strand Breaks and Chromosomal Translocations Induced by DNA Topoisomerase II. Front. Mol. Biosci..

[10] Kobayashi K., Tomita H., Shimizu M., Tanaka T., Suzui N., Miyazaki T., Hara A. (2017). p53 Expression as a Diagnostic Biomarker in Ulcerative Colitis-Associated Cancer. Int. J. Mol. Sci..

[11] Hofseth L.J., Saito S.i., Hussain S.P., Espey M.G., Miranda K.M., Araki Y., Jhappan C., Higashimoto Y., He P., Linke S.P. (2003). Nitric Oxide-Induced Cellular Stress and p53 Activation in Chronic Inflammation. Proc. Natl. Acad. Sci. USA.

[12] Redon C.E., Nakamura A.J., Martin O.A., Parekh P.R., Weyemi U.S., Bonner W.M. (2011). Recent Developments in the Use of γ-H2AX as a Quantitative DNA Double-Strand Break Biomarker. Aging.

[13] Yu T., MacPhail S.H., Banáth J.P., Klokov D., Olive P.L. (2006). Endogenous Expression of Phosphorylated Histone H2AX in Tumors in Relation to DNA Double-Strand Breaks and Genomic Instability. DNA Repair.

[14] Lee Y.-C., Yin T.C., Chen Y.-T., Chai C.-Y., Wang J.Y., Liu M.-C., Lin Y.-C., Kan J.Y. (2015). High Expression of Phospho-H2AX Predicts a Poor Prognosis in Colorectal Cancer. Anticancer Res..

[15] Mirza-Aghazadeh-Attari M., Mohammadzadeh A., Yousefi B., Mihanfar A., Karimian A., Majidinia M. (2019). 53BP1: A Key Player of DNA Damage Response with Critical Functions in Cancer. DNA Repair.

[16] Sharp S.P., Malizia R.A., Walrath T., D’Souza S.S., Booth C.J., Kartchner B.J., Lee E.C., Stain S.C., O’Connor W. (2018). DNA Damage Response Genes Mark the Early Transition from Colitis to Neoplasia in Colitis-Associated Colon Cancer. Gene.

[17] Yang Y.C., Chou H.Y., Shen T.L., Chang W.J., Tai P.H., Li T.K. (2013). Topoisomerase II-Mediated DNA Cleavage and Mutagenesis Activated by Nitric Oxide Underlie the Inflammation-Associated Tumorigenesis. Antioxid. Redox Signal..

[18] Li T.-K., Liu L.F. (2001). Tumor Cell Death Induced by Topoisomerase-Targeting Drugs. Annu. Rev. Pharmacol. Toxicol..

[19] Azarova A.M., Lyu Y.L., Lin C.-P., Tsai Y.-C., Lau J.Y.-N., Wang J.C., Liu L.F. (2007). Roles of DNA Topoisomerase II Isozymes in Chemotherapy and Secondary Malignancies. Proc. Natl. Acad. Sci. USA.

[20] Austin C.A., Lee K.C., Swan R.L., Khazeem M.M., Manville C.M., Cridland P., Treumann A., Porter A., Morris N.J., Cowell I.G. (2018). TOP2B: The First Thirty Years. Int. J. Mol. Sci..

[21] Zhang S., Liu X., Bawa-Khalfe T., Lu L.-S., Lyu Y.L., Liu L.F., Yeh E.T.H. (2012). Identification of the Molecular Basis of Doxorubicin-Induced Cardiotoxicity. Nat. Med..

[22] Fionda C., Abruzzese M.P., Santoni A., Cippitelli M. (2016). Immunoregulatory and Effector Activities of Nitric Oxide and Reactive Nitrogen Species in Cancer. Curr. Med. Chem..

[23] Gao Y., Zhou S., Xu Y., Sheng S., Qian S.Y., Huo X. (2019). Nitric Oxide Synthase Inhibitors 1400W and L-NIO Inhibit Angiogenesis Pathway of Colorectal Cancer. Nitric Oxide.

[24] Meira L.B., Bugni J.M., Green S.L., Lee C.-W., Pang B., Borenshtein D., Rickman B.H., Rogers A.B., Moroski-Erkul C.A., McFaline J.L. (2008). DNA Damage Induced by Chronic Inflammation Contributes to Colon Carcinogenesis in Mice. J. Clin. Investig..

[25] Pereira C., Grácio D., Teixeira J.P., Magro F. (2015). Oxidative Stress and DNA Damage: Implications in Inflammatory Bowel Disease. Inflamm. Bowel Dis..

[26] Hirsch D., Hardt J., Sauer C., Heselmeyer-Hadded K., Witt S.H., Kienle P., Ried T., Gaiser T. (2021). Molecular Characterization of Ulcerative Colitis-Associated Colorectal Carcinomas. Mod. Pathol..

[27] Frick A., Khare V., Paul G., Lang M., Ferk F., Knasmüller S., Beer A., Oberhuber G., Gasche C. (2018). Overt Increase of Oxidative Stress and DNA Damage in Murine and Human Colitis and Colitis-Associated Neoplasia. Mol. Cancer Res..

[28] El Marjou F., Janssen K.-P., Hung-Junn Chang B., Li M., Hindie V., Chan L., Louvard D., Chambon P., Metzger D., Robine S. (2004). Tissue-Specific and Inducible Cre-Mediated Recombination in the Gut Epithelium. Genesis.

[29] Wirtz S., Neufert C., Weigmann B., Neurath M.F. (2007). Chemically Induced Mouse Models of Intestinal Inflammation. Nat. Prot..

[30] Pereira e Silva A., Lourenço A.L., Marmello B.O., Bitteti M., Teixeira G.A.P.B. (2019). Comparison of Two Techniques for a Comprehensive Gut Histopathological Analysis: Swiss Roll versus Intestine Strips. Exp. Mol. Pathol..

[31] Angelou A., Andreatos N., Antoniou E., Zacharioudaki A., Theodoropoulos G., Damaskos C., Garmpis N., Yuan C., Xiao W., Theocharis S. (2018). A Novel Modification of the AOM/DSS Model for Inducing Intestinal Adenomas in Mice. Anticancer Res..

[32] Laubach V.E., Shesely E.G., Smithies O., Sherman P.A. (1995). Mice Lacking Inducible Nitric Oxide Synthase are not Resistant to Lipopolysaccharide-Induced Death. Proc. Natl. Acad. Sci. USA.

[33] Lyu Y.L., Wang J.C. (2003). Aberrant Lamination in the Cerebral Cortex of Mouse Embryos Lacking DNA Topoisomerase IIβ. Proc. Natl. Acad. Sci. USA.

[34] Bianconi F., Kather J.N., Reyes-Aldasoro C.C. (2020). Experimental Assessment of Color Deconvolution and Color Normalization for Automated Classification of Histology Images Stained with Hematoxylin and Eosin. Cancers.

[35] Jensen K., Krusenstjerna-Hafstrøm R., Lohse J., Petersen K.H., Derand H. (2017). A Novel Quantitative Immunohistochemistry Method for Precise Protein Measurements Directly in Formalin-Fixed, Paraffin-Embedded Specimens: Analytical Performance Measuring HER2. Mod. Pathol..

[36] Riley S., Mani V., Goodman M., Dutt S., Herd M. (1991). Microscopic Activity in Ulcerative Colitis: What Does It Mean?. Gut.

[37] Arthur J.C., Perez-Chanona E., Mühlbauer M., Tomkovich S., Uronis J.M., Fan T.-J., Campbell B.J., Abujamel T., Dogan B., Rogers A.B.J.s. (2012). Intestinal Inflammation Targets Cancer-Inducing Activity of the Microbiota. Science.

[38] Chan P.-M., Tan Y.-S., Chua K.-H., Sabaratnam V., Kuppusamy U.R.J.P.o. (2015). Attenuation of Inflammatory Mediators (TNF-α and Nitric Oxide) and Up-Regulation of IL-10 by Wild and Domesticated Basidiocarps of Amauroderma rugosum (Blume & T. Nees) Torrend in LPS-Stimulated RAW264. 7 Cells. PLoS ONE.

[39] Wasserman R.A., Austin C.A., Fisher L.M., Wang J.C. (1993). Use of Yeast in the Study of Anticancer Drugs Targeting DNA Topoisomerases: Expression of a Functional Recombinant Human DNA Topoisomerase II alpha in Yeast. Cancer Res..

[40] Wang Y.-R., Chen S.-F., Wu C.-C., Liao Y.-W., Lin T.-S., Liu K.-T., Chen Y.-S., Li T.-K., Chien T.-C., Chan N.-L. (2017). Producing Irreversible Topoisomerase II-Mediated DNA Breaks by Site-Specific Pt(II)-Methionine Coordination Chemistry. Nucleic Acids Res..

[41] Mao Y., Yu C., Hsieh T.-S., Nitiss J.L., Liu A.A., Wang H., Liu L.F. (1999). Mutations of Human Topoisomerase IIα Affecting Multidrug Resistance and Sensitivity. Biochemistry.

[42] Spitzner J.R., Chung I.K., Muller M.T. (1990). Eukaryotic Topoisomerase II Preferentially Cleaves Alternating Purine-Pyrimidine Repeats. Nucleic Acids Res..

[43] Castro-Dopico T., Dennison T.W., Ferdinand J.R., Mathews R.J., Fleming A., Clift D., Stewart B.J., Jing C., Strongili K., Labzin L.I. (2019). Anti-Commensal IgG Drives Intestinal Inflammation and Type 17 Immunity in Ulcerative Colitis. Immunity.

[44] Geboes K., Riddell R., Öst A., Jensfelt B., Persson T., Löfberg R. (2000). A Reproducible Grading Scale for Histological Assessment of Inflammation in Ulcerative Colitis. Gut.

[45] Rogler G. (2014). Chronic Ulcerative Colitis and Colorectal Cancer. Cancer Lett..

[46] Gorgoulis V.G., Vassiliou L.-V.F., Karakaidos P., Zacharatos P., Kotsinas A., Liloglou T., Venere M., DiTullio R.A., Kastrinakis N.G., Levy B. (2005). Activation of the DNA Damage Checkpoint and Genomic Instability in Human Precancerous Lesions. Nature.

[47] Pommier Y., Nussenzweig A., Takeda S., Austin C. (2022). Human Topoisomerases and Their Roles in Genome Stability and Organization. Nat. Rev. Mol. Cell Biol..

[48] Nitiss J.L. (2009). Targeting DNA Topoisomerase II in Cancer Chemotherapy. Nat. Rev. Cancer.

[49] Pommier Y., Sun Y., Huang S.-y.N., Nitiss J.L. (2016). Roles of Eukaryotic Topoisomerases in Transcription, Replication and Genomic Stability. Nat. Rev. Mol. Cell Biol..

[50] Nitiss J.L. (2009). DNA Topoisomerase II and Its Growing Repertoire of Biological Functions. Nat. Rev. Cancer.

[51] Wang X., Gray Z., Willette-Brown J., Zhu F., Shi G., Jiang Q., Song N.-Y., Dong L., Hu Y. (2018). Macrophage Inducible Nitric Oxide Synthase Circulates Inflammation and Promotes Lung Carcinogenesis. Cell Death Discov..

[52] Feng Z., Cheng Y., Wang Y., Qu S., Du J., Gao F., Liu C., Wang Q., Cai J. (2023). Roxadustat Protect Mice from DSS-Induced Colitis In Vivo by Up-Regulation of TLR4. Genomics.

[53] Jiang N., Wei Y., Cen Y., Shan L., Zhang Z., Yu P., Wang Y., Xu L. (2020). Andrographolide Derivative AL-1 Reduces Intestinal Permeability in Dextran Sulfate Sodium (DSS)-Induced Mice Colitis Model. Life Sci..

[54] Lee S.M., Kim N., Son H.J., Park J.H., Nam R.H., Ham M.H., Choi D., Sohn S.H., Shin E., Hwang Y.J. (2016). The Effect of Sex on the Azoxymethane/Dextran Sulfate Sodium-Treated Mice Model of Colon Cancer. J. Cancer Prev..

[55] Park Y.H., Kim N., Shim Y.K., Choi Y.J., Nam R.H., Choi Y.J., Ham M.H., Suh J.H., Lee S.M., Lee C.M. (2015). Adequate Dextran Sodium Sulfate-Induced Colitis Model in Mice and Effective Outcome Measurement Method. J. Cancer Prev..

[56] Keum N., Giovannucci E. (2019). Global Burden of Colorectal Cancer: Emerging Trends, Risk Factors and Prevention Strategies. Nat. Rev. Gastroenterol. Hepatol..

[57] Pezone A., Olivieri F., Napoli M.V., Procopio A., Avvedimento E.V., Gabrielli A. (2023). Inflammation and DNA Damage: Cause, Effect or Both. Nat. Rev. Rheumatol..

[58] Klusek J., Lewitowicz P., Oblap R., Orlewska E., Witczak B., Marzec M.T., Kozłowska-Geller M., Nawacki Ł., Wawszczak-Kasza M., Kocańda K. (2024). NOS2 Polymorphism in Aspect of Left and Right-Sided Colorectal Cancer. J. Clin. Med..

[59] Alam A., Smith S.C., Gobalakrishnan S., McGinn M., Yakovlev V.A., Rabender C.S. (2023). Uncoupled Nitric Oxide Synthase Activity Promotes Colorectal Cancer Progression. Front. Oncol..

[60] Blackford A.N., Jackson S.P. (2017). ATM, ATR, and DNA-PK: The Trinity at the Heart of the DNA Damage Response. Mol. Cell.

[61] Zanconato F., Battilana G., Forcato M., Filippi L., Azzolin L., Manfrin A., Quaranta E., Di Biagio D., Sigismondo G., Guzzardo V. (2018). Transcriptional Addiction in Cancer Cells is Mediated by YAP/TAZ through BRD4. Nat. Med..

[62] Bradner J.E., Hnisz D., Young R.A. (2017). Transcriptional Addiction in Cancer. Cell.

[63] Davies M., Boyce M., Conway E. (2024). Short Circuit: Transcription Factor Addiction as a Growing Vulnerability in Cancer. Curr. Opin. Struct. Biol..

[64] Ju B.G., Lunyak V.V., Perissi V., Garcia-Bassets I., Rose D.W., Glass C.K., Rosenfeld M.G. (2006). A Topoisomerase IIbeta-Mediated dsDNA Break Required for Regulated Transcription. Science.

[65] Compe E., Egly J.M. (2021). The Long Road to Understanding RNAPII Transcription Initiation and Related Syndromes. Annu. Rev. Biochem..

[66] Linka R.M., Porter A.C., Volkov A., Mielke C., Boege F., Christensen M.O. (2007). C-terminal Regions of Topoisomerase II α and II β Determine Isoform-Specific Functioning of the Enzymes In Vivo. Nucleic Acids Res..

[67] Almeida D., Gerhard R., Leitão D., Davilla C., Damasceno M., Schmitt F. (2014). Topoisomerase II-alfa Gene as a Predictive Marker of Response to Anthracyclines in Breast Cancer. Pathol. Res. Pract..

[68] Basta D.W., Vong M., Beshimova A., Nakamura B.N., Rusu I., Kattah M.G., Shao L. (2023). A20 Restricts NOS2 Expression and Intestinal Tumorigenesis in a Mouse Model of Colitis-Associated Cancer. Gastro. Hep. Adv..

[69] Lee E.G., Boone D.L., Chai S., Libby S.L., Chien M., Lodolce J.P., Ma A. (2000). Failure to Regulate TNF-Induced NF-kappaB and Cell Death Responses in A20-Deficient Mice. Science.

[70] Qu J., Cai Y., Li F., Li X., Liu R. (2025). Potential Therapeutic Strategies for Colitis and Colon Cancer: Bidirectional Targeting STING Pathway. EBioMedicine.

