# Supplementary material for: TOP2 and NOS2 Orchestrate the Generation of DNA Breaks to Promote Colitis Cancer Initiation
Source: Cancers (Basel). 2026 May 8;18(10):1519. doi: 10.3390/cancers18101519 (PMC13205054; doi:10.3390/cancers18101519)
Supplement: Supplementary file 1 [file cancers-18-01519-s001.zip › cancers-4186564-supplementary.pdf]

cancers

MDPI

Article

TOP2 and NOS2 orchestrate the generation of DNA breaks to promote colitis cancer initiation

Ting-Kang Chang, Shiu-Ling Li, Anne-Cécile Brunac, Jia-Jun Huang, Yen-Hsiu Yeh, Pierre Brousset, Jean-Marc Egly, Tsai-Kun Li

1. Supplementary Methods

1.1. The DNA Cleavage and Reversal Assays with Quantitation Analysis

1.1.1. Linear DNA Cleavage and Reversal assays

The reversal assay was performed with an additional reversal step immediately after DNA cleavage reaction. The groups with the same testing compounds were set in parallel, one half was treated with the reversal agent (i.e., EDTA) and the other half was without the reversal agent (i.e., ddH2O). As previously described, the reaction mixture was prepared with linearized pRYG plasmid DNA, TOP2 proteins, and GSNO (63 or 125 µM), in a 1X reaction buffer. The DNA cleavage reaction was performed at 37 ^{0}C for 20 or 30 minutes. Subsequently, 10 mM EDTA (or ddH2O) was added. After 10 min-reversal reaction, proteinase K and SDS were added with a further 60-min incubation at 37 ^{0}C to termination the reactions. Proteolyzed TOP2s were further removed through one round of phenol/chloroform extraction to ensure all the TOP2-bound DNA was released into the liquid samples. The samples were loaded onto a 1% agarose gel in 0.5% Tris-phosphate-EDTA (TPE) for analyses. The DNA fragments were stained with ethidium bromide, visualized under UV light, and photographed using a CCD camera.

1.1.2. Linear DNA Cleavage Assay Quantification

The regions containing smears of DNA fragments below the linear DNA position were quantified through Image J, with removal of the background value of the DNA alone (0%) through subtraction. The remaining values were then normalized against the values of the corresponding TOP2α or TOP2β alone control groups (as 100%).

1.1.3. Linear DNA Cleavage and Reversal Assay with Quantification

The quantification for the reversal ability in the linear DNA cleavage assay is performed as described in the above Section 1.1.2. Briefly, the DNA fragment of each group was first quantified through Image J and converted into percentage (%). The extents of DNA break decrement (i.e., reversal) were calculated through subtraction between the values of the parallel groups with and without the EDTA treatment. Positive values indicate that EDTA reversed the extent of DNA breaks, and thus the greater values represent the higher reversibility.

1.2. Lentivirus-based RNA Interference, Western Blot Analysis and Griess Assay

The Lentivirus-based RNA interference, Western blot analyses, and Griess assay were carried out as previously described [1].

1.2.1. Lentivirus-based RNA Interference

Viral stocks containing non-replicative particles including the lentiviruses vector control (shLuc) and the shTop2β knockdown constructs were purchased from the RNAi core of the Academia Sinica (http://rnai.genmed.sinica.edu.tw). The oligo sequences are listed in the Supplementary Table S2. The infection media was prepared with appropriate volume of the viral stock(s) (5:1, multiplicity of infection), 8 µg/ml polybrene solution, and

DMEM media. 1 x 10^6 HCT116 cells were seed into each well of a 12-well plate. After attachment, the cell growth media was removed and replaced with infection media. After incubated at 37 ^{0}C, 5 % CO2, for 16 hours, the infection media was removed and cells were harvested, then transferred into a 6-well plate with 1 µg/ml puromycin-containing selection media for 3-5 days. The knockdown efficiency was accessed through Western blot analysis.

1.2.2. Western Blot Analysis

HCT116 cells were prepared in DMEM media containing 10 % FBS and 1% P/S. The cells were seeded into a 6-well plate in a density of 1 x 10^6 cells/well. Next day, cells were treated with Etop (50 µM, 1 hour), DSS (5.0%, 4 hours), or GSNO (100 µM, 4 hours). For some experiments, cells were co-treated with ICRF-193 (20 µM), ICRF-187 (100 µM), or PTIO (50 µM). After treatments, cells were lysed using radioimmunoprecipitation (RIPA) lysis and extraction buffer (Thermo Scientific, Waltham, MA, USA). After separated by sodium dodecyl sulfate-polyacrylamide gel electrophoresis (SDS-PAGE), the proteins were transferred onto nitrocellulose membranes in a Mini Trans-Blot Cell (Bio-Rad) overnight. The quality of the proteins in the membranes were determined using Ponceau S staining. The membrane was blocked with 5% non-fat milk in 1X TBS-T at RT (50 rpm, 2 hours), and probed with diluted primary antibodies (RT, 50 rpm, 2 hours). After washed with 1X TBS-T (RT, 100 rpm, 10 minutes; 3 times), the membranes were incubated with diluted secondary antibodies for an additional hour with shaking (50 rpm) at RT. The membranes were further washed with 1X TBS-T three times, reacted with Western Lightening Plus ECL (PerkinElmer, Shelton, CT, USA) in dark for additional 3 minutes. Then, the signals were detected using a Kodak M-35A X-OMAT Automatic Processor system. The primary antibodies used were rabbit γH2AX pAbs (1:2500, Genetax), rabbit NOS2 pAbs (1:5000, Millipore), rabbit TOP2β pAbs (1:1000, Santa Cruz), rabbit α-Actin 1 pAb (1:10000, Abcam), mouse GAPDH pAbs (1:10,000, Genetex), and the secondary antibodies used were goat anti-rabbit IgG horseradish peroxidase (HRP) (1:10,000, Bethyl) or goat anti-mouse IgG HRP (1:10,000, Abnova, Taipei, Taiwan).

1.2.3 Griess Assay

5 x 10^5 RAW264.7 cells (~ 80% confluency) were seeded into each well with DMEM media containing 10% FBS and 1% penicillin and streptomycin (P/S) in a 12-well plate. After overnight cultivation in a CO_2 Incubator, the cell culture medium was removed, and the cells were washed with 1X PBS buffer. For stimulation, 500 ml of Phenol-red free RPMI media containing 1 mg/ml of LPS and 100 U/ml of IFNγ was added into each well and further incubated at 37 ^{0}C for 4–8 hours. The media and cells were separated and subjected to either Griess or Western blot analyses. Griess analysis was conducted by mixing equal volumes of the media with Griess reagent (Sigma). The reaction mixture was incubated in the dark for 15 minutes and then transferred into a 96-well microtiter plate. The absorbance of the colorimetric product was measured at 570 nm using a Biochrom WPA spectrophotometer (Biochrom Ltd., Waterbeach, Cambridge, UK). The quantity of nitrite production was determined based on the formula of absorbance = 0.0044 mM of $NO2^-$. This formula used sodium nitrate for calibration.

2. Supplementary Figures

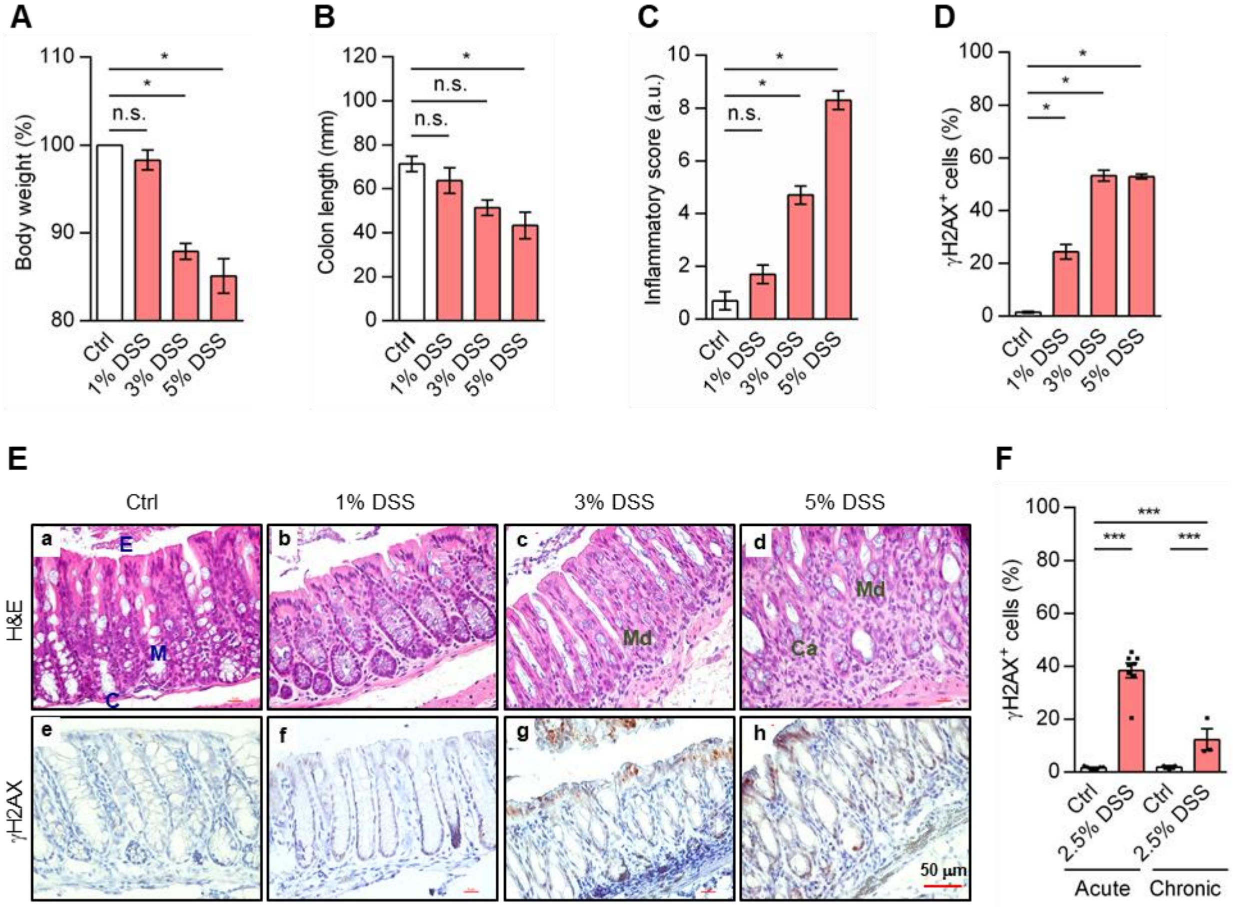

Supplementary Figure S1. DNA damage responses and pathological features of mice after DSS treatments. Female mice, 6-8 weeks old, were administered 1%, 3%, or 5% DSS in ddH_2O for 5 days to induce acute colitis (one repetition, *n* = 3). (**A**-**C**) Body weight was expressed as a percentage of the ddH2O control (Ctrl) group, while colon length and inflammatory score were evaluated according to described criteria. (**D**) The percentage of $\gamma H2AX^+$ cells was determined through manual cell counting (> 100 cells per image, > 2 images per sample; see Methods). (**E**) H&E and IHC images of the DSS treated female mice. After 5 days of treatment, mice were euthanized, and sections of colon tissues were stained with H&E (panels a-d) and IHC (panels e-h) with γH2AX antibodies. H&E staining revealed normal epithelial layer (E), mucin (M) and crypt structures (C) in control tissue samples (Ctrl). In the acute DSS-treated colon specimens, the corresponding pathologic structures of crypt abscess (Ca) and mucin depletion (Md) are indicated. (**F**) Populations of $\gamma H2AX^+$ cells (%) of male acute and chronic colitis groups were determined. The data were obtained from the corresponding experiments (as shown in Figure 1), but quantitated by manual cell counting (acute DSS 38.4 ± 2.8%, two repetitions, *n* = 7-8; chronic DSS 12.3 ± 4.1%, one repetition, *n* = 3). Statistical analyses were conducted using one-way ANOVA with Tukey multiple comparisons. Each dot represents a quantitated result from one mouse. Error bars = SEM; *, $P \leq 0.05$; ***, $P \leq 0.001$; a.u., arbitrary unit.

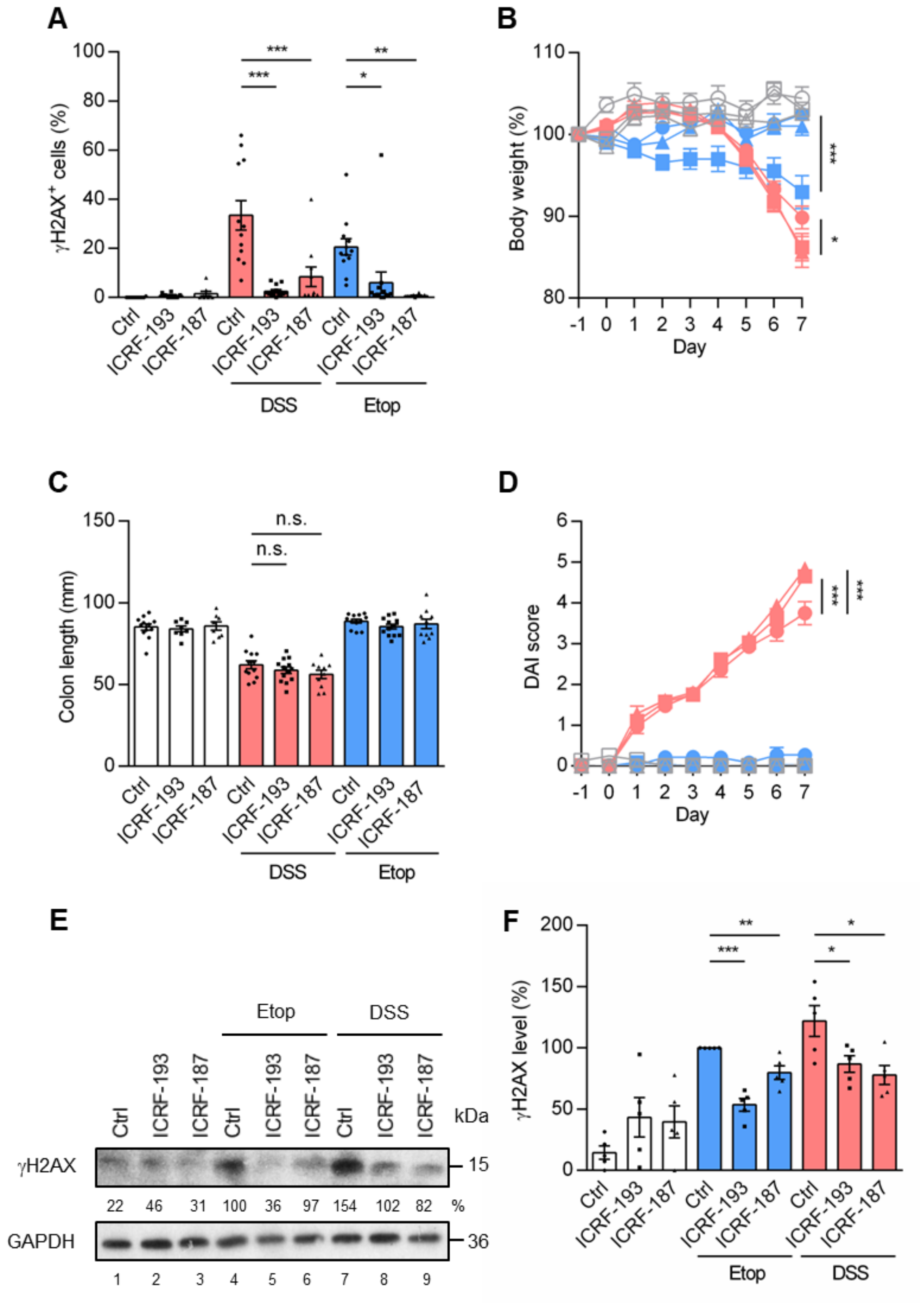

Supplementary Figure S2. TOP2-antagonizing inhibitors on the DSS-UC or etoposide-treated mice. The mice were treated with ICRF-193 (2.5 mg/kg) and ICRF-187 (15 mg/kg). After 1 day, mice were received either 7 days of DSS (2.5%) or Etop (40 mg/kg) treatments) (three repetitions, $n \geq 8$). The pathological features were determined and scored. (**A**) Effects of ICRF on γH2AX+ cell populations were quantitated by manual cell counting (> 100 cells per image, > 2 images per sample; see Methods; DSS = 33.4 + 6.0, + ICRF-193 = 2.5 + 0.6, + ICRF-187 = 8.4 + 4.0%; Etop = 20.5 + 3.3, + ICRF-193 = 6.0 + 4.4, + ICRF-187 = 0.7 + 0.2%), while (**B**) body weight, (**C**) colon length, and (**D**) DAI activity were investigated. Ci◯e ( ): Ctrl; Trian△e ( ): ICRF-187; Squ□e ( ): ICRF-193. (**E**) HCT116 cells were treated with Etop (50 μM, 1 hour) or DSS (5.0%, 4 hours) with or without co-treatments of ICRFs, lysed and then subjected to Western blot analysis for detection of γH2AX. (**F**) Quantification of γH2AX detection was performed via Image J, with normalization against GAPDH, and re-leveled based on the Etop control group (five repetitions, $n = 5$). Statistical analyses were conducted using one-way ANOVA with Tukey multiple comparisons (**A, C**), two-way ANOVA with Sidak

multiple comparisons (**B, D**) or two-tailed unpaired *t*-test (**F**). Each dot represents a quantitated result from one mouse. ICRF-193; Red: DSS treatment; Blue: Etop treatment; *, $P \leq 0.05$; **, $P \leq 0.01$; ***, $P \leq 0.001$; n.s., non-significant.

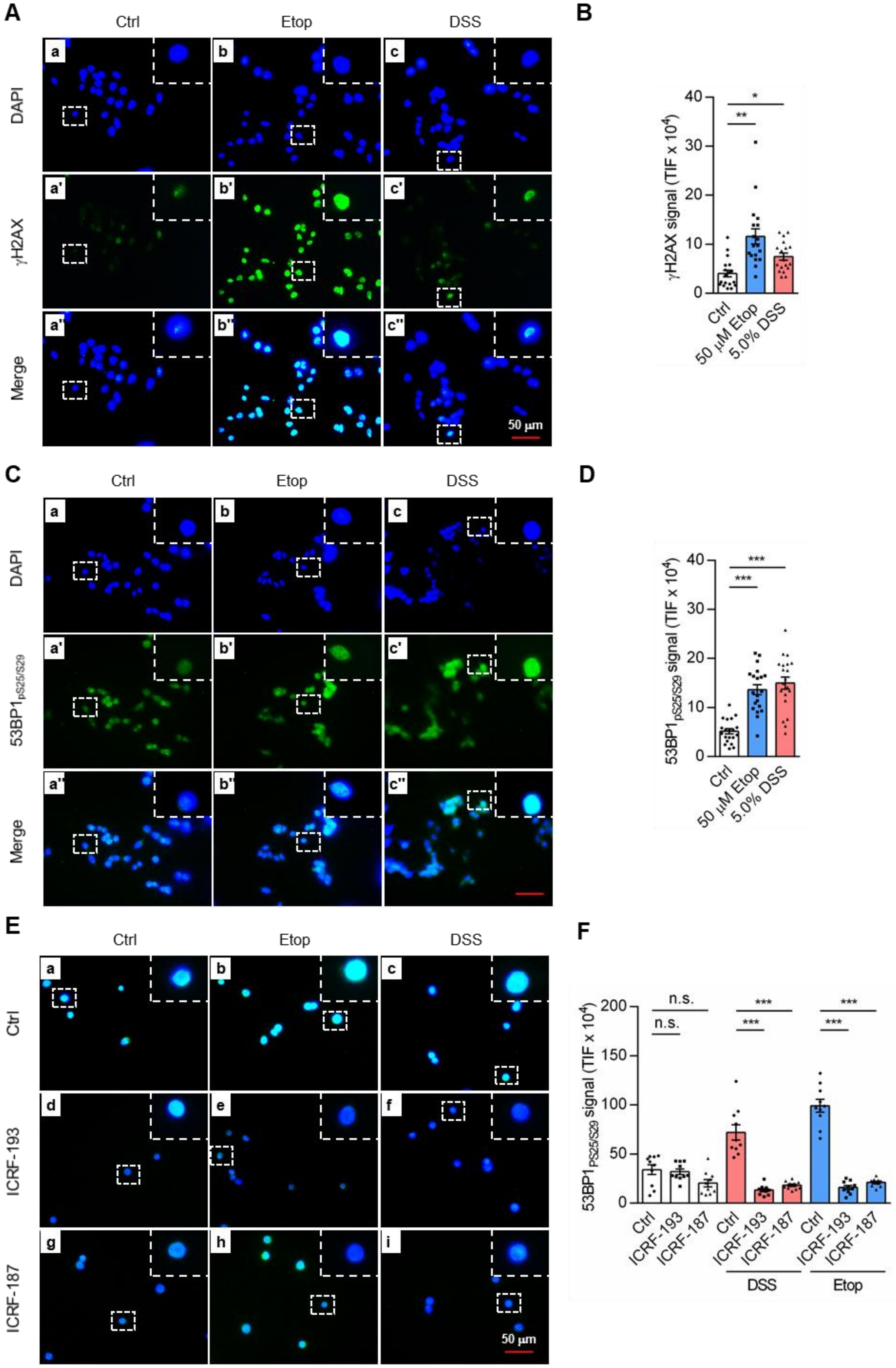
A
Ctrl
Etop
DSS
DAPI
γH2AX
Merge
a
b
c
a'
b'
c'
a''
b''
c''
50 µm
B
γH2AX signal (TIF x 10^4)
Ctrl
50 µM Etop
5.0% DSS
C
Ctrl
Etop
DSS
DAPI
53BP1pS25/S29
Merge
D
53BP1pS25/S29 signal (TIF x 10^4)
Ctrl
50 µM Etop
5.0% DSS
E
Ctrl
Etop
DSS
Ctrl
ICRF-193
ICRF-187
a
b
c
d
e
f
g
h
i
50 µm
F
53BP1pS25/S29 signal (TIF x 10^4)
n.s.
Ctrl
ICRF-193
ICRF-187
DSS
Etop

Supplementary Figure S3. 53BP1 phosphorylation under etoposide and DSS treatments. HCT116 cells grown on cover slides were treated with DSS (5.0%, 4 hours) or Etop (50 μM, 1 hour). When indicated on each side of the panels, ICRFs were co-treated to the cells with either Etop or DSS. After treatments, cells were subjected to immuno-fluorescence assays. Detections of (**A**) γH2AX and (**C**) $53BP1_{pS25/S29}$ (400X image; 1000X, dash square of the magnified inset) in HCT116 cells, and quantitative results of (**B**) γH2AX (three repetitions, n = 18) and (**D**) $53BP1_{pS25/S29}$ (three repetitions, n = 20). Effects of ICRF-187/193 on both (**E**) Etop- and DSS-induced $53BP1_{pS25/S29}$ (the merged images, 400X image; 1000X, dash square**)** and (**F**) quantitative results (three repetitions, n = 10). As indicated above, at least ten cells per group were used for quantification. Statistical analyses were conducted using one-way ANOVA with Tukey multiple comparisons. Each dot represents a quantitated result from one cell. TIF: total immuno fluorescence; *, $P \le 0.05$; **, $P \le 0.01$; ***, $P \le 0.001$; n.s., non-significant.

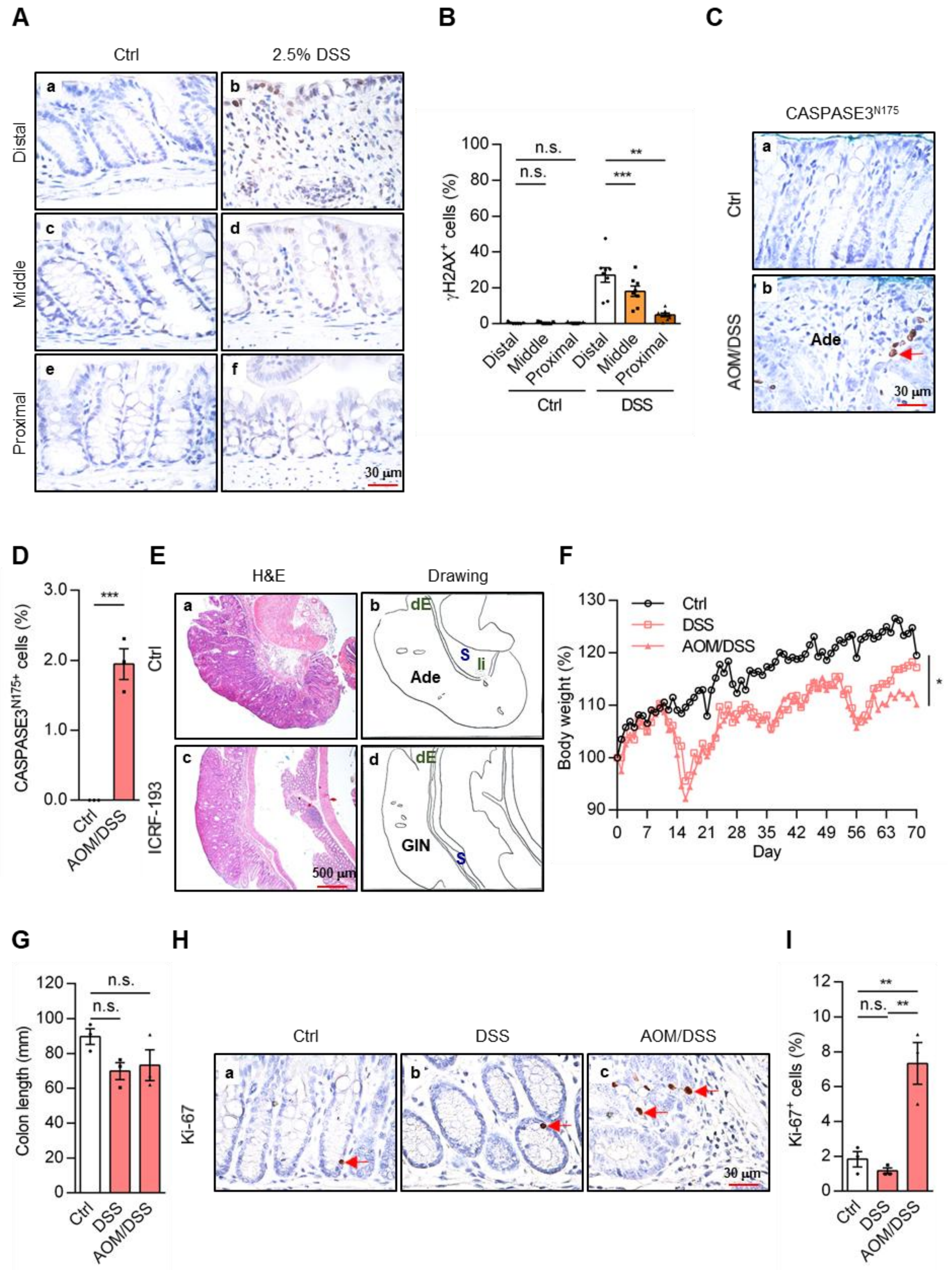

140

Supplementary Figure S4. The effect of ICRF-193 on tumor progression in the colon cancer disease model. (**A**) Relative population of γH2AX⁺ cells at the distal, middle and proximal segments of each colon and (**B**) quantitative results (with 2.5% DSS; distal = 27.1 ± 4.0, middle = 18.1 ± 2.9, proximal = 4.9 ± 1.0%; two repetitions, *n* ≥ 7) via manual cell counting as described above. (**C**) Apoptotic caspase 3^{N175} in AOM/DSS-induced tumors and (**D**) quantitative results via manual cell counting (one repetition, *n* = 3). (**E**) H&E staining of a tumor located in the distal colon of untreated (Ctrl; panel a, 141 142 143 144 145 146

drawing in panel b) and ICRF-193-treated mice (panel c, drawing in panel d) in the AOM/DSS-induced cancer model. Normal submucosa (S), adenoma (Ade), gastrointestinal neoplasia (GIN), dysplastic epithelium (dE), and infiltrated immune cells (Ii) are indicated. (**F**) Daily body weight and (**G**) colon length (measured at day 70**)** of mice in the chronic colitis and cancer models (one repetition, $n = 3$). (**H**) IHC staining of Ki-67 staining of mouse colon samples in the chronic colitis and cancer models with (**I**) quantitation (Ctrl = 1.8 ± 0.4, DSS = 1.2 ± 0.2, AOM/DSS = 7.3 ± 1.2%; one repetitions, $n = 3$) by manual cell counting. Statistical analyses were conducted using two-tailed paired *t*-test (**D**), one-way ANOVA with Tukey multiple comparisons (**G**, **I**), or two-way ANOVA with Sidak multiple comparisons (**B**, **F**). Each dot represents a quantitated result from one mouse. *, $P \leq 0.05$; **, $P \leq 0.01$; ***, $P \leq 0.001$; n.s., non-significant.

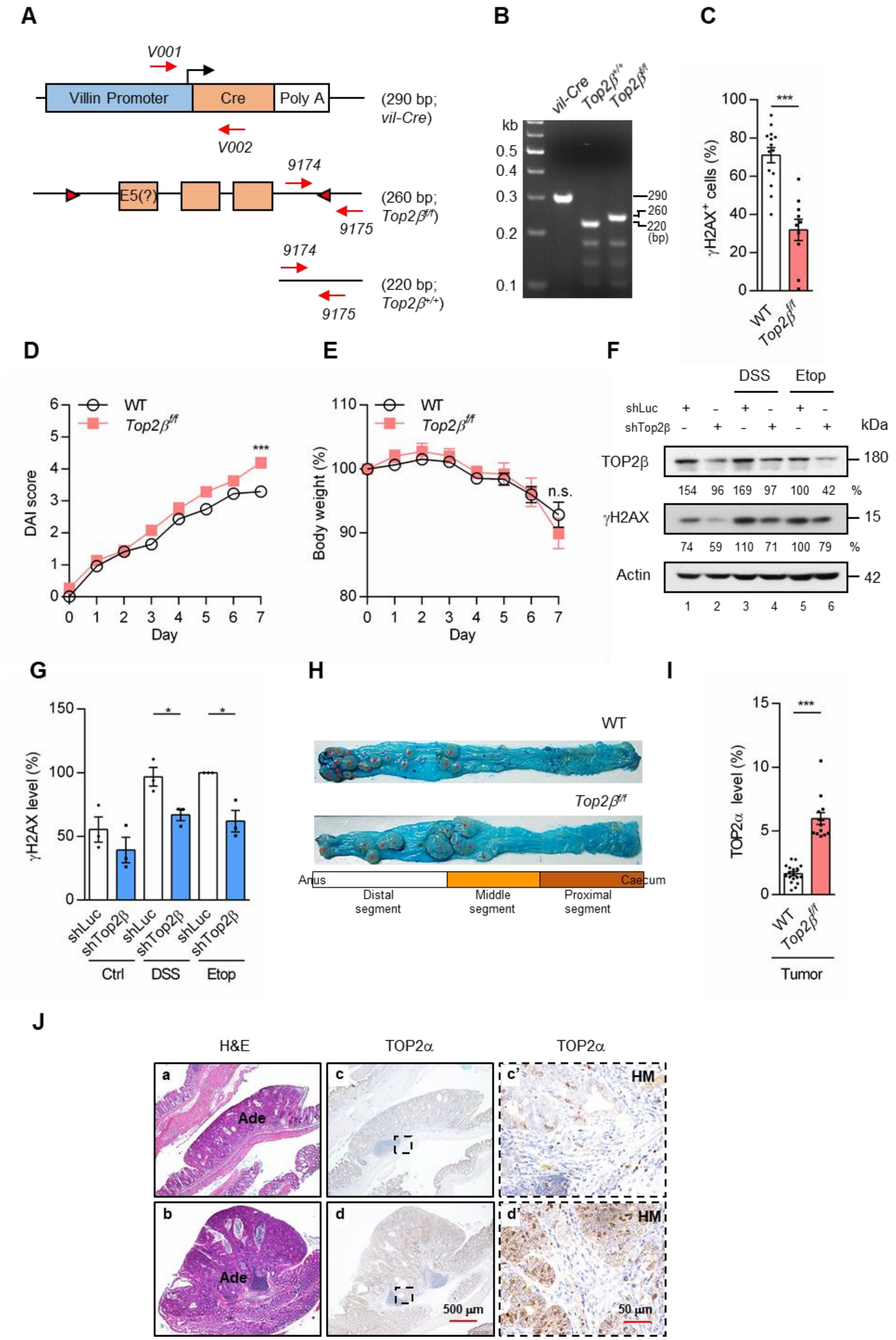

A
V001
Villin Promoter
Cre
Poly A
(290 bp; vil-Cre)
V002
9174
E5(?)
(260 bp; Top2β^f/f)
9175
9174
(220 bp; Top2β^+/+)
9175
B
vil-Cre
Top2β^+/+
Top2β^f/f
kb
0.5
0.4
0.3
0.2
0.1
290
260
220
(bp)
C
γH2AX⁺ cells (%)
***
WT
Top2β^f/f
D
WT
Top2β^f/f
DAI score
Day
E
Body weight (%)
n.s.
Day
F
DSS
Etop
shLuc
shTop2β
kDa
TOP2β
180
154 96 169 97 100 42 %
γH2AX
15
74 59 110 71 100 79 %
Actin
42
1 2 3 4 5 6
G
γH2AX level (%)
shLuc
shTop2β
Ctrl
DSS
Etop
H
WT
Top2β^f/f
Anus
Caecum
Distal segment
Middle segment
Proximal segment
I
TOP2α level (%)
Tumor
J
H&E
TOP2α
Ade
HM
500 μm
50 μm

Supplementary Figure S5. Generating a colon adenoma disease model with the *vil-Cre* $Top2\beta^{f/f}$ colonic conditional knockout mice. (**A**) PCR-based genotyping of *vil-Cre*-containing, WT ($Top2\beta^{+/+}$) and $Top2\beta^{f/f}$ mice. (**B**) Schematic illustration of the mouse gene constructs and primers. Primers *V001* and *V002* targeted the promoter and *Cre* gene, respectively. The $Top2\beta^{f/f}$ construct contained two *loxP* sites flanking the three exons encoding the active core with tyrosine. The forward (*9174*) and reverse (*9175*) primers targeted the flanking intronic regions of the second *loxP* site (red triangle). (**C**) $\gamma H2AX^+$ cells quantitated by manual cell counting (> 100 cells per image, 2 images per sample), (**D**) DAI activity, and (**E**) daily body weight of WT and $Top2\beta^{f/f}$ mice treated with 2.5% DSS for 7 days to induce acute colitis (four repetitions, $n \geq 10$). (**F**) Protein levels of TOP2β paralleled with extents of Etop- and DSS-induced γH2AX expression. The shTop2β knockdown cell line was established with the lentiviral system and two cell lines were treated with DSS (5.0%, 4 hours) or Etop (50 μM, 1 hour). Western Blot analysis was applied to detect expression levels TOP2β and γH2AX. (**G**) Quantification analysis of the extents of γH2AX and TOP2β expression was done through Image J with normalization against GAPDH and then re-leveled with the Etop control groups (three repetitions, $n = 3$). (**H**) Images of tumor visualized in the three segments. Red dots indicate tumor location**.** (**I**) Expression of TOP2α in WT and $Top2\beta^{f/f}$ mouse colons in the AOM/DSS cancer setting compared to those of WT mice (five repetitions, $n \geq 13$). (**J**) H&E and IHC images of TOP2α staining in the cancer disease model. Statistical analyses were conducted using two-tailed unpaired *t*-test (**C**, **G, I**) or two-way ANOVA with Sidak multiple comparisons (**D**, **E**). Each dot represents a quantitated result from one mouse. HM: high magnification; $^*P \leq 0.05$; $^{***}P \leq 0.001$; n.s., non-significant.

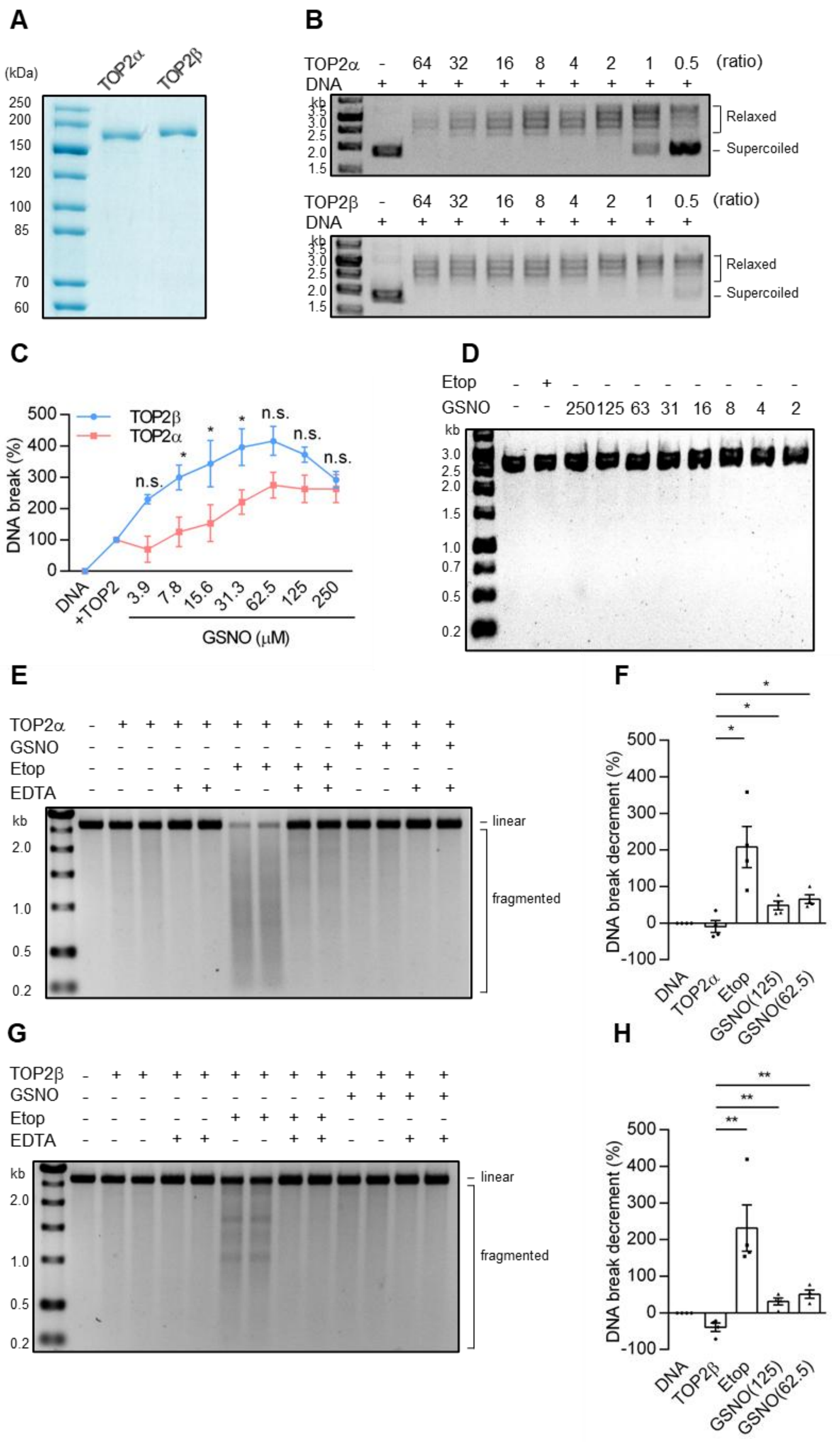

Supplementary Figure S6. Nitric oxide activated TOP2α- and TOP2β- mediated DNA breaks. (**A**) Coomassie blue staining of purified human recombinant TOP2α and TOP2β. (**B**) DNA relaxation

assays of increasing amounts of TOP2α and TOP2β using pRYG plasmid as DNA substrate with incubation at 30 ^{o}C for 30 min. The molar ratio range tested was from 0.5 to 64 (TOP2/DNA). (**C**) NO induced DSBs in the presence of TOP2β over TOP2α (e.g., 31.3 μM GSNO: TOP2β = 396.3 + 58.3% > TOP2α = 220.6 + 38.9%; three repetitions, n = 3). (**D**) The GNSO DNA binding assay was conducted by incubating the linearized pRYG plasmid with GNSO (2-250 μM.) at 37 ^{0}C for 30 min, followed by one-hour incubation in 1% SDS and 1 mg/ml protease K. Etop (50 μM) was used as a positive control. DNA cleavage and reversal assays were performed in the presence of recombinant (**E**) TOP2α (63 μM GSNO; quantitative results of the EDTA reversibility in (**F**); four repetitions, n = 4) and (**G**) TOP2β isozymes (16 μM GSNO; quantitative results of the EDTA reversibility in (**H**); four repetitions, n = 4) for 20 (even lanes) or 30 min (odd lanes) and quantitated as described in Methods. Etop (50 μM) was used as a positive control. A higher value (%) indicates a greater reversibility. Statistical analyses were conducted using two-way ANOVA with Sidak multiple comparisons (**C**) or two-tailed unpaired t-test (**F**, **H**). Each dot represents a quantitated result from one cleavage reversal reaction. *, $P \leq 0.05$; **, $P \leq 0.01$; ***, $P \leq 0.001$; n.s., non-significant.

195

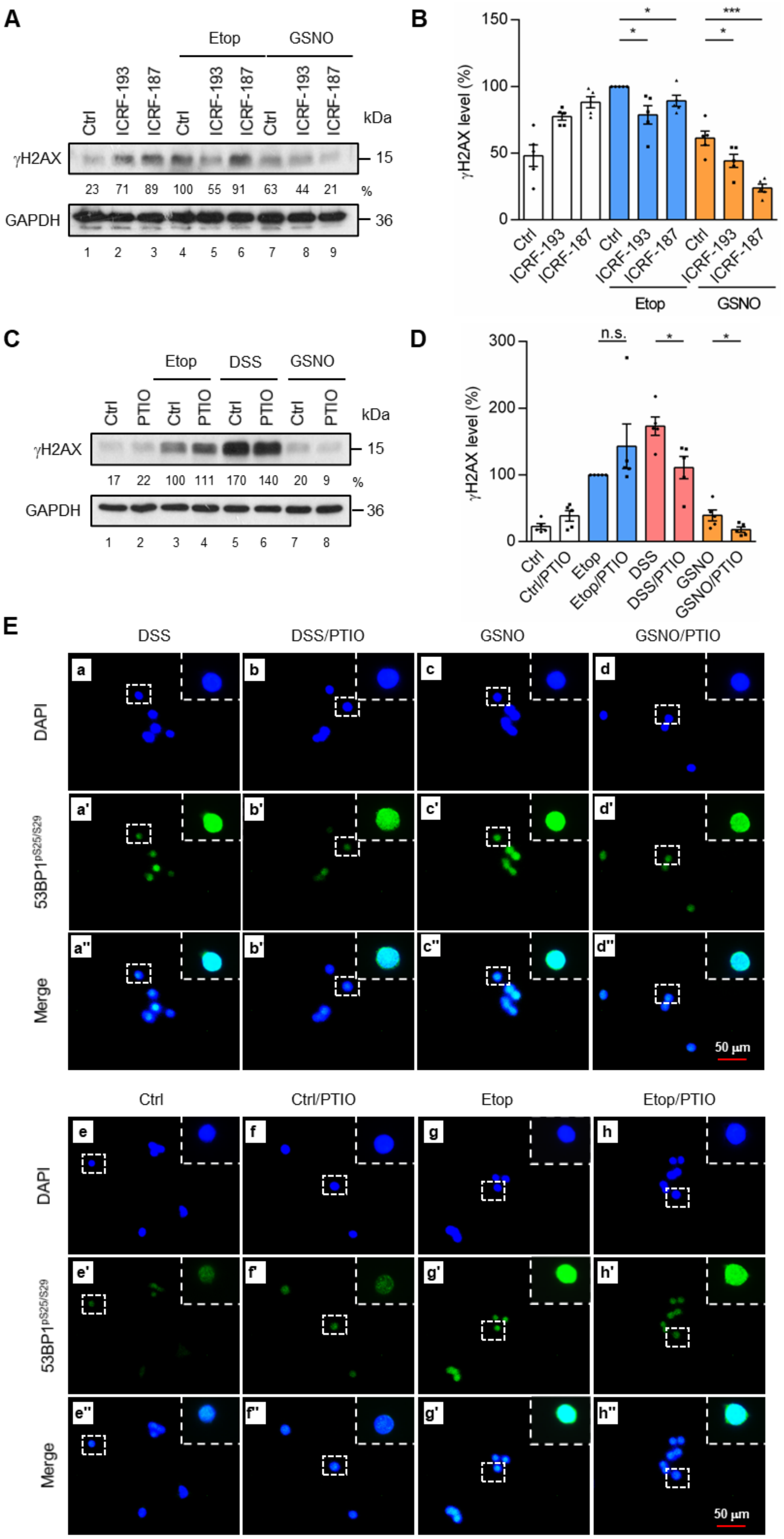
A
Etop
GSNO
Ctrl
ICRF-193
ICRF-187
kDa
γH2AX
15
23 71 89 100 55 91 63 44 21 %
GAPDH
36
1 2 3 4 5 6 7 8 9
B
γH2AX level (%)
Etop
GSNO
C
DSS
PTIO
17 22 100 111 170 140 20 9 %
D
n.s.
Ctrl/PTIO
Etop/PTIO
DSS/PTIO
GSNO/PTIO
E
DAPI
53BP1pS25/S29
Merge
50 µm

Supplementary Figure S7. Both DSS and GSNO treatments induced γH2AX and 53BP1$_{pS25/S29}$ detections. (**A**-**B**) ICRF-193 and ICRF-187 co-treatment reduced both Etop- and GSNO-induced γH2AX expression. HCT116 cells were treated with Etop (50 μM, 1 hour) or GSNO (100 μM, 4 hours) in the presence or absence of ICRFs co-treatments. (**A***)* Western blot analysis was carried out to detect the levels of γH2AX expression, (**B**) and quantitative results (five repetitions, *n* = 5; through Image J, normalized against GAPDH, and re-leveled based on the Etop control group). (**C**-**E**) DSS- and GSNO-, but not Etop-induced γH2AX and 53BP1$_{pS25/S29}$ detections were reduced with the co-treatment of a NO scavenger PTIO. Cells were divided into 8 groups for treatments as indicated at the top of panels (50 μM PTIO, 1 or 4 hours of co-treatment; 50 μM Etop, 1 hour; 5.0% DSS, 4 hours; 100 μM GSNO, 4 hours; various combinations) and then subjected to (**C**) Western blot of γH2AX with (**D**) quantitative results (five repetitions, *n* = 5), and (**E**) immunofluorescence analyses (53BP1$_{pS25/S29}$; 400X image; 1000X, dash square of the magnified inset). Statistical analyses were conducted using two-tailed unpaired *t*-test (**B, D**). Each dot represents a quantitated result from one cleavage reversal reaction. *$P \leq 0.05$; ***$P \leq 0.001$; n.s., non-significant.

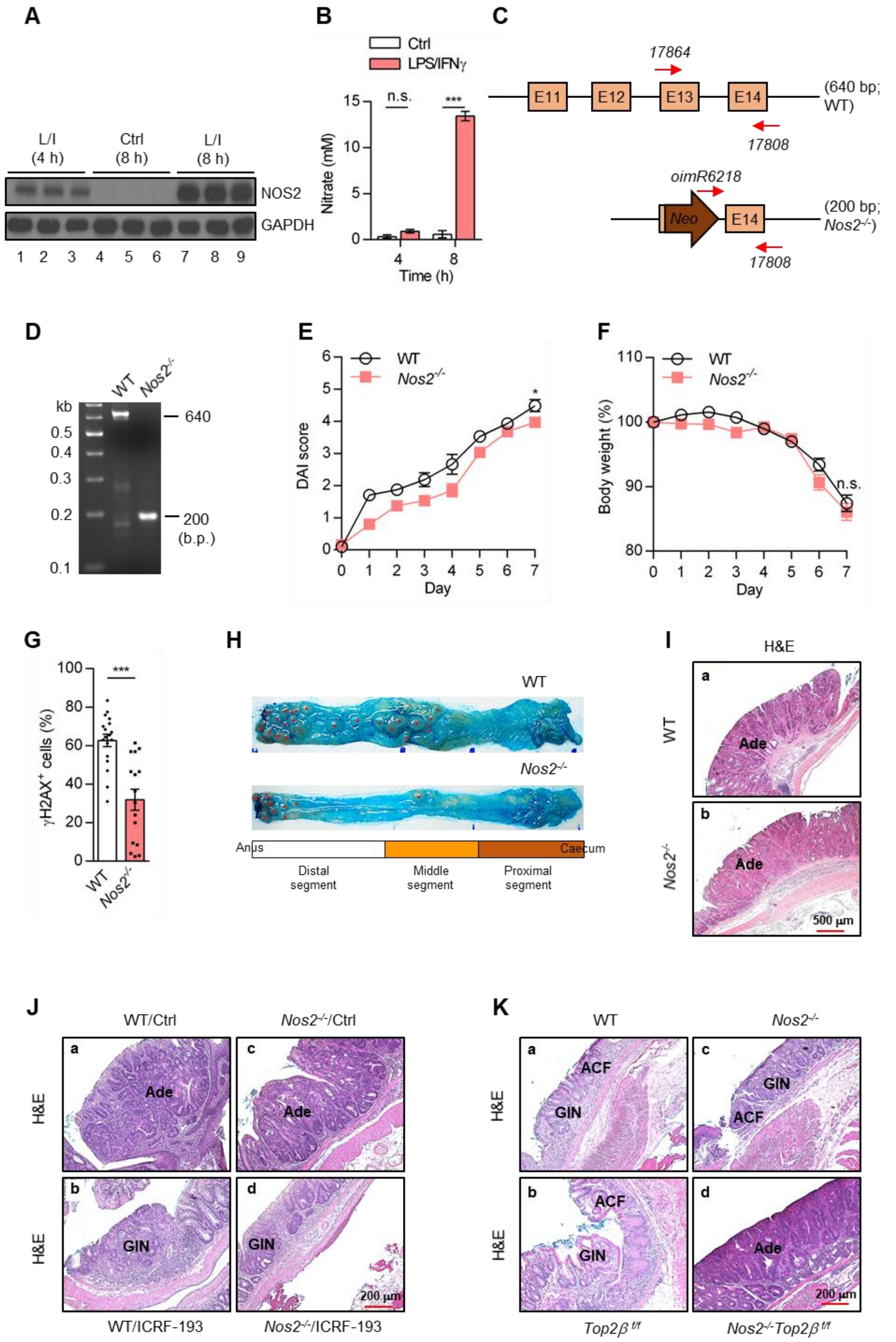
A
L/I (4 h)
Ctrl (8 h)
L/I (8 h)
NOS2
GAPDH
1 2 3 4 5 6 7 8 9
B
Ctrl
LPS/IFNγ
n.s.
***
Nitrate (mM)
Time (h)
C
17864
E11
E12
E13
E14
(640 bp; WT)
17808
oimR6218
Neo
E14
(200 bp; Nos2-/-)
17808
D
WT
Nos2-/-
kb
640
200
(b.p.)
E
WT
Nos2-/-
DAI score
Day
F
WT
Nos2-/-
Body weight (%)
n.s.
Day
G
γH2AX+ cells (%)
***
WT
Nos2-/-
H
WT
Nos2-/-
Anus
Caecum
Distal segment
Middle segment
Proximal segment
I
H&E
WT
Nos2-/-
Ade
500 μm
J
WT/Ctrl
Nos2-/-/Ctrl
H&E
Ade
GIN
200 μm
WT/ICRF-193
Nos2-/-/ICRF-193
K
WT
Nos2-/-
ACF
GIN
Ade
200 μm
Top2β f/f
Nos2-/-Top2β f/f

Supplementary Figure S8. Nos2 deficiency caused a reduction in DAI score, but not in body-weight and tumor growth/progression. Raw264.7 cells were treated with LPS and IFNγ (L/I) for the indicated times. (**A**) NOS2 expression and (**B**) NO production was determined (three repetitions; *n* = 3). (**C**) PCR-based genotyping of the *Nos2* gene defective mice. (**D**) Schematic drawing of the mouse NOS2 gene constructs and primers used. In the *Nos2*$^{-/-}$ construct, a neomycin resistant gene (*Neo*; dark brown arrow) was inserted and replaced with three exons (E11-E13) which encoded for the NOS2 calmodulin domain. The forward (*17864*, *oimR6218*) and reverse (*17808*) primers targeted the gene sequences of E13, Neo, and E14. (**E**) DSS-induced body weight, (**F**) DAI activity and (**G**) populations of γH2AX^{+} cells in WT (four repetitions, *n* = 18) and Nos2-/- mice (*n* = 16). Each dot represents a quantitated result from one mouse. (**H**) Representative images of tumors in the colons of WT and *Nos2*$^{-/-}$ mice stained with Alcian blue and (**I**) of tumor sections with the H&E staining. (**J**) Representative tumor images of in WT and *Nos2*$^{-/-}$ mice with AOM/DSS cancer setting with either ICRF-193 treatment or (**K**) additional *Top2β*$^{f/f}$ genetic deficiency. Statistical analyses were conducted using two-way ANOVA with Sidak multiple comparisons (**B**, **E**, **F**) or two-tailed unpaired *t*-test (**G**). ACF, aberrant crypt foci; GIN, gastrointestinal neoplasia; Ade, Adenoma; *, $P \leq 0.05$; ***, $P \leq 0.001$; n.s., non-significant.

Supplementary Table S1. Primer sequences for mouse genotyping.

Primers for *Nos2* site (Jax code: 10894)	
oIMR6218	5′-CCT TCT ATC GCC TTC TTG ACG-3′
17864	5′-TCC GAT TTA GAG TCT TGG TGA-3′
17808	5′-TCA CCA CCA GCA GTA GTT GC-3′
Primers for *vil-Cre* site [2]	
V001	5′-CAA GCC TGG CTC GAC GGC C-3′
V002	5′-CGC GAA CAT CTT CAG GTT CT-3′
Primers for *Top2β* site (Jax code: 23605)	
9174	5′-GCC TTC CCC TTT CAC TGT CT-3′
9175	5′-GCT TGG GAA TTG TTT GCT GT-3′

Supplementary Table S2. The shRNA Sequences for Lentivirus Knockdown of Luciferase and TOP2β

Name	Oligo Sequence
shLuc976	CCGGGCGGTTGCCAAGAGGTTCCATCTCGAGATGGAACCTCTT-GGCAACCGCTTTTTG
shTop2β	CCGGGTAGAGCCTGAGTGGTATATTCTCGAGAATATACCACTCAGGCTC-TACTTTTTG

Supplementary Table S3. Antibodies.

Name	Source	Identification
Rabbit polyclonal γH2AX	Genetex	GTX127340, RRID:AB_2885642
Mouse monoclonal γH2AX	Abcam	AB26350, RRID:AB_470861
Rabbit monoclonal CASPASE3N175	CST	9579, RRID:AB_10897512
Rabbit polyclonal NOS2	Millipore	AB5382, RRID:AB_91825
Rat monoclonal F4/80	Cedarlane	CL8940AP, AB_10060355
Rabbit monoclonal Ki-67	Abcam	AB16667, AB_302459
Rabbit polyclonal 53BP1$_{pS25/S29}$	Bioss	BS-3020R, RRID:AB_10856138
Rabbit monoclonal, TOP2α	Abcam	AB52934, RRID:AB_883143
Rabbit polyclonal TOP2β	Santa Cruz	SC13059, AB_2205866
Rabbit polyclonal α-Actin 1	Abcam	A2066, AB_476693
Mouse monoclonal GAPDH	Genetex	GTX627408, AB_2888014
Anti-rabbit universal polymer	Nichirei Biosciences	14141F
Anti-rabbit IgG HRP	Bethyl	A120-101P, RRID:AB_67264
Anti-rabbit Alexa Fluor 488	Invitrogen	A11034, RRID:AB_2576217
Anti-mouse IgG-Fc-HRP	Bethyl	A110-128P, RRID:AB_67545
Anti-mouse IgG-HRP	Abnova	PAB0096, RRID:AB_1575355

Supplementary Table S4. Special chemicals and reagents.

Name	Source	Identification
Azoxymethane (AOM)	Sigma	A5486
Dextran Sodium Sulfate (DSS)	MP Biomedicals	9011-18-1
Etoposide (VP-16)	ENZO	BML-GR307
ICRF-193	ENZO	BML-GR332
Dexrazoxane (ICRF-187)	Abcam	AB141109
Dimethyl sulfoxide (DMSO)	Sigma	D8418
Alcian Blue	Sigma	A5268
Hematoxylin	Leica	3801560
Eosin	Leica	3801600
Citric Acid Monohydrate	Sigma	C1909
Trisodium Citrate Dehydrate	Sigma	S1804
Trypsin	Sigma	T7409
CC1 Antigen Retrieval Buffer	Ventana Medical	950-124

Hydrogen Peroxide (H2O2)	Riedel-de Haen	31642
Bovine serum albumin (BSA)	Sigma	A9418
Penicillin-Streptomycin (PS)	Thermo Fisher	15140122
MyTaq™ HS Red Mix	Bioline	BIO-2504
Puromycin	Sigma	P8833
Polybrene	Sigma	TR1003
Lipopolysaccharides (LPS)	Sigma	L4005
Interferonγ (IFNγ)	Sigma	I4777
Protease inhibitor	Roche	11873580001
Griess Reagent	Sigma	G4410
Sodium Nitrate	Sigma	7631-99-4
RIPA Lysis and Extraction Buffer	Thermo	89900
Ponceau S	Sigma	P7170
Western Lightening Plus ECL	PerkinElmer	NEL104001EA
Coomassie Brilliant blue G250	Thermo	20279
Hind III-HF endonuclease	NEB	R3104L
S-nitrosoglutathione (GSNO)	Sigma	N4148
Carboxyl-PTIO	Cayman Chemical	81540
Paraformaldehyde	Sigma	P6148
Fluroshield with DAPI	Genetex	GTX30920
Adenosine Triphosphate (ATP)	BioBasic	AB0020
Dithiotheitol (DTT)	Millipore	114740001
Proteinase K	Bioshop	PRK403

Supplementary Table S5. Bacteria, yeast and cell growth media. 232

Name	Source	Identification
DMEM	Thermo Fisher	11965092
RPMI Phenol-Red Free	Thermo Fisher	11835030
Fetal Bovine Serum (FBS)	Thermo Fisher	16000069
CSM-U broth	[3]	
Terrific broth (TB)	Thermo Fisher	A1374301

Supplementary Table S6. Critical commercial assays. 233

Name	Source	Identification
Hemoccult SENSA Developer kit	Beckman Coulter	60152
DAB Peroxidase Substrate Kit	ImmPACK	SK-4105
OmniMap DAB anti-Rb detection kit	Roche	05266548001
MyTaq gDNA Extract Kit	Bioline	BIO-21127
PureLink™ HiPure Plasmid Maxiprep Kit	Invitrogen	K210006

Supplementary Table S7. Cell lines, bacteria, yeast, Lentiviruses, mouse strains. 234

Name	Source	Identification
HCT116	ATCC	
RAW264.7 cell	ATCC	
DH5α_pRYG *E. coli*	Addgene	113642
BCY123_YEpWob6 (hTOP2α, the first 28 of the 1531 aa were replaced by the first 5 codons of yeast TOP2	[4]	Obtained from Prof. James C. Wang
BCY123_YEphTOP2β (hTOP2β)	[5]	Obtained from Prof. Leroy F. Liu
Lentivirus TRC2 shLuc976	Academia Sinica RNAi Core	TRCN0000231719

Lentivirus NM_001068.2-2554s21c1 (shTop2β)	Academia Sinica RNAi Core	TRCN0000233296
C57BL/6Jnarl	NAR	
B6.Cg-Tg(*Vil-cre*)20SyNci	Frederick National Lab	01XE7
129S $Top2\beta^{tm2.1(vil\text{-}cre)Jcw}$/J	The Jackson Laboratory	
B6. 129P2-$Nos2^{tm1/Lau}$/J	The Jackson Laboratory	002609
B6; 129S $Top2\beta^{tm2.1(vil\text{-}cre)Jcw}$/J	This lab breeding	
B6; 129S-$Top2\beta^{tm2.1(vil\text{-}cre)Jcw}Nos2^{tm1Lau}$/J	This lab breeding	

References

1. Yang, Y.C.; Chou, H.Y.; Shen, T.L.; Chang, W.J.; Tai, P.H.; Li, T.K. Topoisomerase II-Mediated DNA Cleavage and Mutagenesis Activated by Nitric Oxide Underlie the Inflammation-Associated Tumorigenesis. *Antioxid. Redox Signal.* **2013**, *18*, 1129-1140, doi:10.1089/ars.2012.4620.
2. El Marjou, F.; Janssen, K.-P.; Hung-Junn Chang, B.; Li, M.; Hindie, V.; Chan, L.; Louvard, D.; Chambon, P.; Metzger, D.; Robine, S. Tissue-Specific and Inducible Cre-Mediated Recombination in the Gut Epithelium. *Genesis* **2004**, *39*, 186-193, doi:10.1002/gene.20042.
3. Wang, Y.-R.; Chen, S.-F.; Wu, C.-C.; Liao, Y.-W.; Lin, T.-S.; Liu, K.-T.; Chen, Y.-S.; Li, T.-K.; Chien, T.-C.; Chan, N.-L. Producing Irreversible Topoisomerase II-Mediated DNA Breaks by Site-Specific Pt(II)-Methionine Coordination Chemistry. *Nucleic Acids Res.* **2017**, 10861-10871, doi:10.1093/nar/gkx742.
4. Wasserman, R.A.; Austin, C.A.; Fisher, L.M.; Wang, J.C. Use of Yeast in the Study of Anticancer Drugs Targeting DNA Topoisomerases: Expression of a Functional Recombinant Human DNA Topoisomerase II alpha in Yeast. *Cancer Res.* **1993**, *53*, 3591-3596.
5. Mao, Y.; Yu, C.; Hsieh, T.-S.; Nitiss, J.L.; Liu, A.A.; Wang, H.; Liu, L.F. Mutations of Human Topoisomerase IIα Affecting Multidrug Resistance and Sensitivity. *Biochemistry* **1999**, *38*, 10793-10800, doi:10.1021/bi9909804.

Disclaimer/Publisher's Note: The statements, opinions and data contained in all publications are solely those of the individual author(s) and contributor(s) and not of MDPI and/or the editor(s). MDPI and/or the editor(s) disclaim responsibility for any injury to people or property resulting from any ideas, methods, instructions or products referred to in the content.
